# Pyroptosis of oral keratinocyte contributes to energy metabolic reprogramming of T cells in oral lichen planus via OPA1-mediated mitochondrial fusion

**DOI:** 10.1038/s41420-024-02174-1

**Published:** 2024-09-17

**Authors:** Zaiwu Yang, Miao Deng, Lin Ren, Zhaona Fan, Shiwen Yang, Suyang Liu, Xianyue Ren, Jinlong Gao, Bin Cheng, Juan Xia

**Affiliations:** 1 Hospital of Stomatology, Guanghua School of Stomatology, Sun Yat-sen University, No.56 Lingyuan Xi Road, Yuexiu District, 510055 Guangzhou, P. R. China; 2 Guangdong Provincial Key Laboratory of Stomatology, No.74 Zhongshan Second Road, Yuexiu District, 510055 Guangzhou, P. R. China; 3 Sydney Dental School, Faculty of Medicine and Health, The University of Sydney, Institute of Dental Research, Westmead Centre for Oral Health, Westmead, 2145 Australia

**Keywords:** Cell biology, Cell death, Glycobiology, Inflammation

## Abstract

Oral lichen planus (OLP) is a chronic inflammatory disease that is associated with an increased risk of carcinogenesis. The typical pathological features of OLP include submucosal T-cell banding, infiltration, and liquefactive degeneration of basal epithelial cells. However, the histological appearance of basal cell death cannot be explained by apoptosis of keratinocytes alone. The aim of this study was to explore a novel mechanism of epithelial cell death, pyroptosis, and its role in the development of OLP. The immunohistochemical results initially revealed pyroptosis in the epithelial cells of OLP. There was significant upregulation of pyroptosis-related inflammatory cytokines, specifically IL-1β. The expression of IL-1β is closely related to the severity of the patient’s condition. In vitro, the culture supernatant from epithelial cells and exogenous IL-1β significantly promote the proliferation and activation of T cells. This effect can be inhibited by neutralizing antibody or receptor inhibitor of IL-1β. Stimulation with exogenous IL-1β enhances both glycolysis and oxidative phosphorylation in T cells, with a more pronounced increase in glycolysis. This is due to the regulation of NAD^+^ availability and mitochondrial dynamics by IL-1β. IL-1β specifically stimulates the expression of optic atrophy 1 (OPA1), particularly L-OPA1, which promotes mitochondrial fusion and increases NAD^+^ availability. This process upregulated glycolysis in T cells. The knockdown of OPA1 reverses these changes by reducing the proliferation and activation of T cells. In this study, IL-1β promoted OPA1 transcription by activating the NF-κB pathway. The expression of OPA1 is inhibited by the inhibitor of NF-κB pathway. These results suggest that OLP keratinocytes undergo pyroptosis, which then secrete inflammatory factors that activate the NF-κB signaling pathway of T cells. This pathway regulates OPA1-mediated mitochondrial fusion and energy metabolism reprogramming in T cells, contributing to the development of OLP. These findings provide new insights into the mechanisms and therapeutic strategies for OLP.

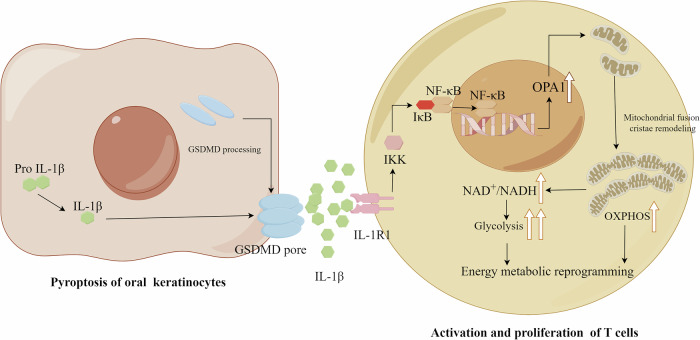

## Introduction

Oral lichen planus (OLP) is a chronic inflammatory disease of oral mucosa. As one of the most common potentially malignant oral diseases, OLP poses an increased risk of carcinogenesis. The global prevalence rate of OLP is estimated to be between 0.49% and 1.43%, with a malignant transformation rate of approximately 1.14% [1–3]. However, the pathogenesis of OLP remains incompletely understood.

The most characteristic pathological changes associated with OLP are the banding of T lymphocytes in the lamina propria adjacent to the epithelium and liquefaction of basal cells. Activated T cells attacking epithelial cells are one of the pathogenic mechanisms of OLP, leading to the death of OLP epithelial basal cells [1, 2]. Apoptosis is the process of programmed cell death in keratinocytes. Colloid bodies at the junction between the basal layer and lamina propria are considered apoptotic basal cells under a light microscope [3]. However, the characteristics of apoptotic cells, such as shrinking cell volume, pyknosis, and integrity of the cell membrane structure, cannot explain the vacuolar and liquefaction degeneration of basal cells in OLP under electron microscopy. The mitochondria and rough endoplasmic reticulum in basal cells are swollen, and basal cells are dissolved and broken when vacuoles appear in the cytoplasm [4]. These findings indicate the possibility of additional cell death pathways beyond apoptosis.

Pyroptosis is a crucial innate immune response that plays a significant role in combating infections and endogenous risks. This response gradually releases cell contents until the cell membrane ruptures, triggering a robust inflammatory response [5]. The activation of caspase-1, GSDMD cleavage, and the release of IL-1β and IL-18 are important markers of pyroptosis. Pyroptosis occurs faster than apoptosis and is accompanied by the release of several proinflammatory factors [6]. The elevated level of NLRP3 inflammatory corpuscles in OLP lesions suggests a correlation between pyroptosis and OLP [7]. However, a direct relationship between OLP and pyroptosis has yet to be discovered.

T lymphocyte proliferation and activation require significant ATP energy. Glycolysis and OXPHOS are the primary energy sources that synergistically and competitively maintain cellular energy balances [8, 9]. In the resting state, T cells are primarily energized by OXPHOS. However, activated T cells may rely more on glycolysis, which produces ATP faster than OXPHOS does. This ensures that activated T cells can grow and differentiate rapidly in response to the antigen receptor on their surface [10]. Previous studies have also found that T cells exhibit a metabolic form of aerobic glycolysis, similar to the Warburg effect [11]. However, the role of reprogramming T-cell energy metabolism in OLP remains to be explored.

Mitochondria are highly dynamic organelles that reshape T-cell metabolism through mitochondrial fusion/fission and cristae remodeling, influencing T-cell function and fate [12]. Effector T cells exhibit increased mitochondrial mass and ATP production, which contribute to immune response activation and cytokine secretion [13]. Several proteins regulate mitochondrial fission and fusion. Dynamin-related protein 1 (DRP1) binds to adapter proteins such as mitochondrial fission 1 (Fis1) and mitochondrial fission factor (MFF) to mediate mitochondrial fission. Optic atrophy 1 (OPA1) anchors to the inner mitochondrial membrane (IMM) and regulates mitochondrial fusion along with mitofusin (MFN) proteins on the outer mitochondrial membrane [14]. Disruption of the balance between fusion and fission has been linked to various diseases [15]. Mitochondrial fragmentation is frequently observed in resting cells, whereas fused and networked mitochondria assist high-energy-demand cells in meeting their energy needs [16, 17]. Cellular metabolic reprogramming is coordinated by changes in the mitochondrial dynamics.

Based on the question of the death pattern of basal cells, we found that the pathogenic factors may lead to pyroptosis of keratinocytes in OLP. The inflammatory factor IL-1β, released from pyroptosis, activates the NF-κB pathway of T cells, promoting mitochondrial fusion mediated by OPA1 and participating in the reprogramming of T-cell energy metabolism. Activated T cells cause cell death in epithelial cells. These findings provide new insights for developing preventive and therapeutic strategies that target pyroptosis to inhibit the occurrence and development of OLP.

## Results

### Increased expression of pyroptosis-related proteins is closely related to the disease severity of patients with OLP

**Fig. 1:**
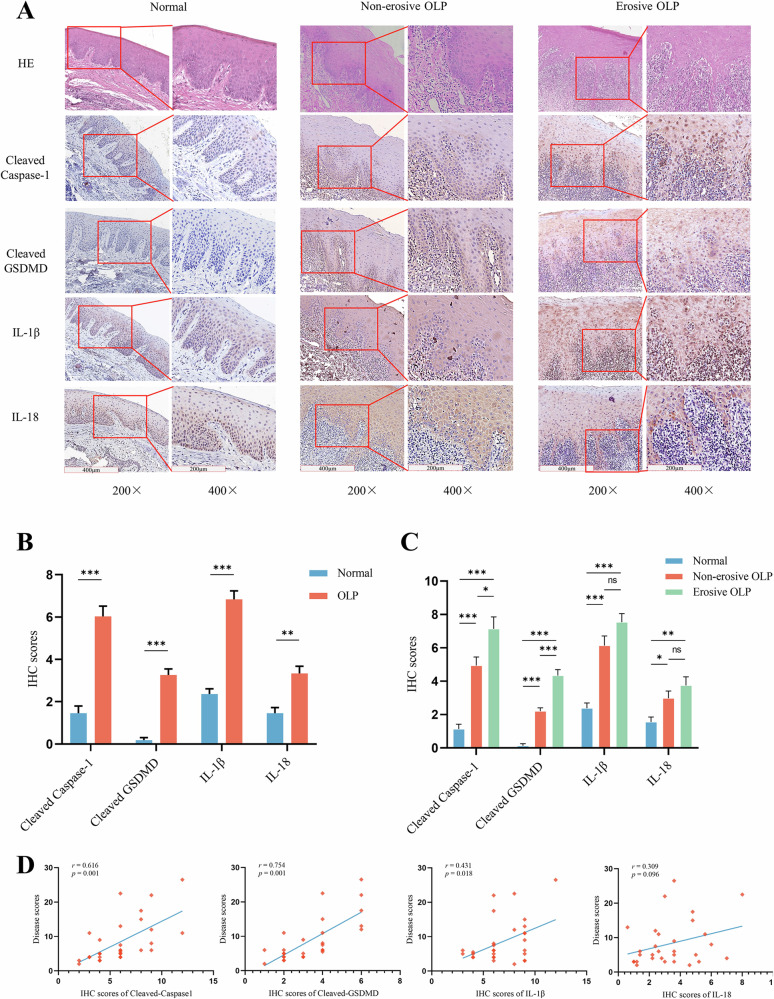
Increased expression of pyroptosis-related proteins in OLP oral epithelial tissues is closely related to the severity of patients’ conditions. **A** H&E staining and IHC assays for pyroptosis-related proteins in mucosal tissues. Sample sizes: Normal: *n* = 10, OLP: *n* = 30. **B**, **C** IHC semiquantitative analysis of pyroptosis-related protein expression in mucosal epithelial tissues. **D** Pearson correlation analysis for correlation between the expression levels of pyroptosis-related proteins in mucosal epithelial tissues and severity of OLP. *P < 0.05. **P < 0.01. ***P < 0.001. H&E hematoxylin and eosin, IHC immunohistochemical assays.

To investigate pyroptosis in OLP epithelial cells, we analyzed the expression of pyroptosis-related proteins in 30 OLP and 11 normal oral mucosal tissues. IHC showed that the expression of pyroptosis-related proteins, such as cleaved caspase-1, cleaved GSDMD, IL-18, and IL-1β, in the OLP epithelial layer was significantly higher than that in normal oral mucosal tissues, primarily expressed in the spinous and basal layers. Caspase-1 is cleaved and expressed mainly in the cytoplasm and nucleus, whereas GSDMD is cleaved and is predominantly expressed in the cell membrane and cytoplasm. IL-18 is primarily expressed in the cytoplasm. It is noteworthy that IL-1β was highly expressed not only in the spinous and basal layers but also in the connective tissue of the lamina propria (Fig. 1A, B). Compared with normal oral mucosal tissue, erosive OLP showed increased levels of pyroptosis-related proteins. Specifically, levels of cleaved caspase-1 and cleaved GSDMD were significantly upregulated (Fig. 1C). These findings suggest that pyroptosis occurs in the epithelial cells of the OLP.

To investigate the correlation between the expression of pyroptosis-related proteins and clinical conditions in patients with OLP, we conducted Pearson’s correlation analysis to examine the relationship between their expression levels in 30 OLP tissue samples and reticulation/keratosis, erythema, and ulceration (REU) scores (Supplementary Table S1). A significant positive correlation was found between the expression of cleaved caspase-1, cleaved GSDMD, and IL-1β in OLP tissues and REU scores (correlation coefficients of 0.626, *P* < 0.001; 0.754, *P* < 0.001; and 0.431, *P* = 0.018, respectively) (Fig. 1D). However, IL-18 levels did not significantly correlate with OLP progression (*r* = 0.431, *P* = 0.096). Given the specific distribution of IL-1β in the OLP tissues, pyroptosis of epithelial cells could affect the T cells of the lamina propria through the release of proinflammatory cytokines, such as IL-1β. The expression of IL-1β significantly correlated with the severity of OLP.

### IL-1β, pyroptosis-released inflammatory factor, enhances T-cell proliferation, and activation

**Fig. 2:**
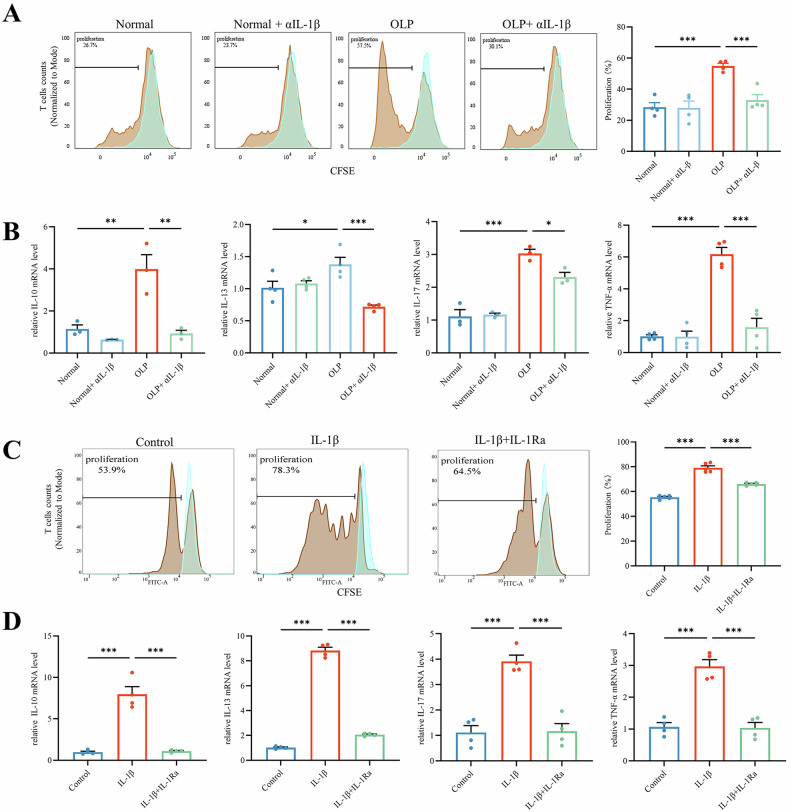
IL-1β, pyroptosis-released inflammatory factor, enhances T-cell proliferation and activation. **A** CellTrace™ CFSE-labeled OLP-T cells conditioned co-cultured with culture supernatant from indicated primary epithelial cells, analyzed using flow cytometry, proliferation rates of OLP-T cells in the indicated groups were quantitatively analyzed. The blue represents the primary peak from co-culturing with fresh epithelial cell culture medium, without CD3/CD28 stimulation, and the brown represents the proliferation peak under the indicated conditions. **B** The mRNA levels of inflammatory factors released after T-cell activation, detected after conditioned co-culturing with culture supernatant from indicated primary epithelial cells. **C** CellTrace™ CFSE-labeled OLP-T cells analyzed using flow cytometry, under treatment with exogenous IL-1β, with or without IL-1Ra, proliferation rates were quantitatively analyzed. The blue represents the primary peak without CD3/28 stimulation, the brown represents the proliferation peak under the indicated conditions. **D** The mRNA levels of inflammatory factors released after T-cell activation, under treatment with exogenous IL-1β, with or without IL-1Ra. *P < 0.05. **P < 0.01. ***P < 0.001. OLP-T cells T cells from peripheral blood of patients with OLP, αIL-1β IL-1β neutralizing antibody; IL-1Ra inhibitor of IL-1β receptor 1.

To investigate the effects of IL-1β released during pyroptosis on T cells, we isolated primary oral keratinocytes from healthy individuals and patients with OLP. These cells were then co-cultured with peripheral blood T lymphocytes from patients with OLP. The culture supernatant from OLP epithelial cells significantly increased T-cell proliferation, with a notably higher proliferation rate than that in the normal group (Fig. 2A). Multiplying T cells had high levels of activation markers such as IL-10, IL-13, IL-17, and TNF-α (Fig. 2B). The neutralizing antibody against IL-1β significantly reduced both the rate of cell multiplication and the pyroptosis-related proteins cytokines in T cells stimulated by epithelial cells (Fig. 2A, B). These results suggest that IL-1β, which is released by pyroptotic epithelial cells in OLP, is a key factor in promoting T-cell proliferation and activation.

Subsequently, we used exogenous IL-1β to validate its effects on T lymphocytes. We discovered that IL-1R1, the receptor for IL-1, was expressed in lymphocytes in the lamina propria of the OLP tissue and peripheral blood T lymphocytes (Supplementary Fig. S1A, B). Stimulation of T cells from patients with OLP (OLP-T cells) with exogenous IL-1β results in a significant increase in cell proliferation and activation. This was characterized by a marked leftward shift in the proliferation peak (Fig. 2C) and increased the expression of activating factors (Fig. 2D). IL-1Ra, an IL-1β receptor inhibitor, reduced the proliferation peak of T cells and attenuated the upregulation of activating factors (Fig. 2C, D). These findings highlight the important role of IL-1β in promoting T-cell proliferation and activation in OLP.

### IL-1β regulates energy metabolism reprogramming of T cells in OLP

**Fig. 3:**
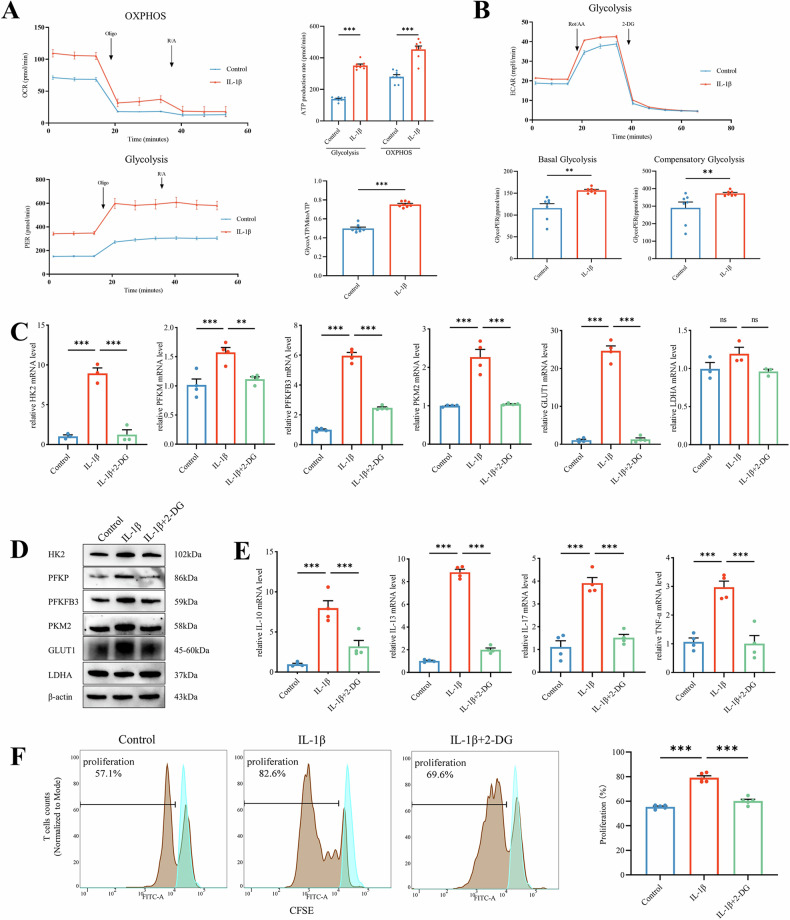
IL-1β regulates energy metabolism reprogramming of T cells in OLP. **A** Seahorse^®^ analysis of ATP production rate and source changes in OLP-T cells showed variations in the PER and OCR over time, quantification by Agilent Seahorse Wave. **B** Seahorse ^®^ analysis of the glycolysis rate in OLP-T cells revealed variations in the ECAR over time, quantification by Agilent Seahorse Wave. **C**, **D** The mRNA and protein levels of glycolysis-related genes in OLP-T cells under indicated treatment. **E** The mRNA levels of inflammatory factors released after T-cell activation, under treatment with exogenous IL-1β, with or without 2-DG. **F** CellTrace™ CFSE-labeled OLP-T cells analyzed using flow cytometry, under the treatment with exogenous IL-1β, with or without 2-DG, proliferation rates were quantitatively analyzed. The blue represents the primary peak without CD3/CD28 stimulation, the brown represents the proliferation peak under the indicated conditions. *P < 0.05. **P < 0.01. ***P < 0.001. OLP-T cells T cells from peripheral blood of patients with OLP, 2-DG 2-deoxy-D-glucose, PER proton efflux rate, OCR oxygen consumption rate, ECAR extracellular acidification rate.

To investigate the effect of IL-1β on T cells, we measured the proton efflux rate (PER) and oxygen consumption rate (OCR) to evaluate the changes in ATP levels. Our study found that IL-1β increased both aerobic glycolysis and OXPHOS activities in OLP-T cells, resulting in a higher proportion of ATP derived from glycolysis (Fig. 3A). The Extracellular acidification rate (ECAR) serves as an indicator of glycolytic activity [18]. ECAR analysis showed that IL-1β-treated OLP-T cells exhibited stronger basal and compensatory glycolysis (Fig. 3B), indicating a higher glycolytic activity. The proportion of energy produced by OXPHOS during the basal phase also decreased (Supplementary Fig. S2A). IL-1Ra also reduced the glycolysis-promoting effect of IL-1β, suppressing the ECAR of OLP-T cells (Supplementary Fig. S2B).

We confirmed changes in glycolytic activity by analyzing the mRNA and protein levels of key glycolytic enzymes in OLP-T cells under IL-1β stimulation. IL-1β stimulates the expression of glucose transporter 1 (GLUT1), hexokinase 2 (HK2), phosphofructokinase (PFK), pyruvate kinase M2 (PKM2), and 6-phosphofructo-2-kinase/fructose-2,6-biphosphatase 3 (PFKFB3). The classic glycolysis inhibitor 2-deoxy-D-glucose (2-DG) significantly inhibited this effect (Fig. 3C, D and Supplementary Fig. S2C). However, IL-1β did not increase the expression of lactate dehydrogenase A (LDHA) (Fig. 3C and Supplementary Fig. S2C).

2-DG suppressed the effect of IL-1β on the upregulation of glycolysis-related genes (Fig. 3C, D and Supplementary Fig. S2C). It inhibited the shift in the ATP production ratio toward glycolysis in T cells, as expected (Supplementary Fig. S2D). Further studies revealed that OLP-T cells costimulated with 2-DG and IL-1β did not show significant proliferation compared to those stimulated with IL-1β alone. The expression of T-cell activation-related factors was also lower (Fig. 3E, F). These results indicated that IL-1β promotes the proliferation and activation of OLP-T cells by regulating glycolytic metabolic reprogramming.

### IL-1β enhances L-OPA1 expression and mitochondrial fusion, increasing NAD^+^ availability in T cells

**Fig. 4:**
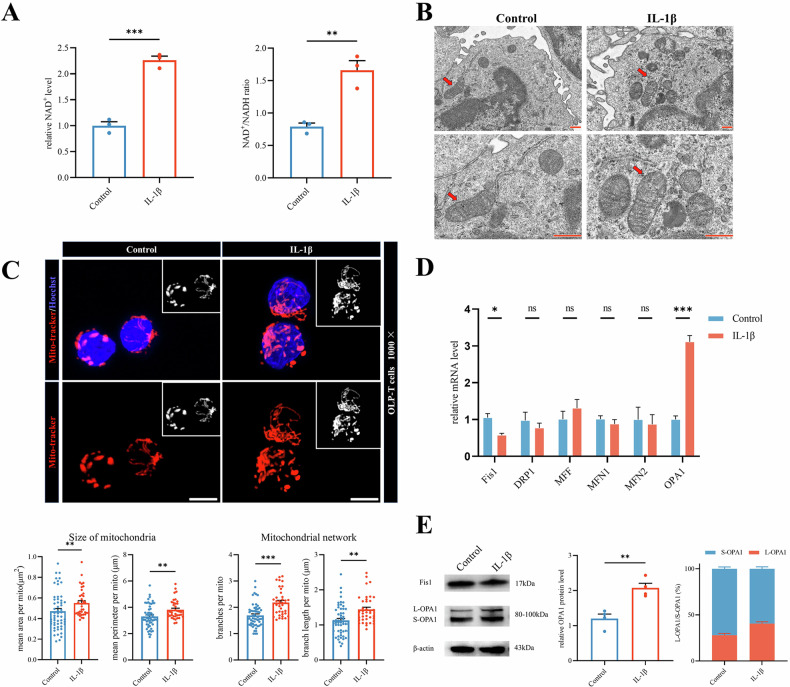
IL-1β enhances L-Opa1 expression and mitochondrial fusion, increasing NAD^+^ availability in T cells. **A** NAD^+^ content and NAD^+^/NADH ratio in OLP-T cells stimulated with exogenous IL-1β, detected by NAD^+^ Assay Kit. **B** Mitochondria of OLP-T cells captured by transmission electron microscopy after IL-1β treatment, with mitochondria indicated by red arrows, bar: 500 nm. **C** Mitochondria in OLP-T cells labeled with Mitotracker captured by confocal microscopy after IL-1β treatment, bar: 5 μm. Mitochondria labeled with Mitotracker, highlighted in red, and cell nuclei labeled with Hoechst, depicted in blue. Confocal images were analyzed using Image J to assess the size and network structure of mitochondria. **D** The mRNA levels of Mitochondrial dynamics-related genes in OLP-T cells. **E** The protein levels of OPA1 and Fis1 in OLP-T cells, analyzed by Image J. *P < 0.05. **P < 0.01. ***P < 0.001. OLP-T cells T cells from peripheral blood of patients with OLP.

What is the mechanism by which IL-1β promotes T-cell glycolysis? We investigated the role of NAD^+^ in glycolysis, tricarboxylic acid cycle, and OXPHOS-driven energy metabolism [19]. We found an increase in NAD^+^ levels and the NAD^+^/NADH ratio in OLP-T cells under IL-1β stimulation (Fig. 4A).

NAD^+^ activity is regenerated through respiratory chain complexes and mitochondrial shuttle systems, which are closely correlated with mitochondrial dynamics [20–22]. Mitochondrial morphology can control metabolic alterations in T cells [12]. Therefore, we inferred that NAD^+^ activity and metabolism were affected by mitochondrial dynamics. We further examined the mitochondrial morphology of OLP-T cells. Using TEM, we observed remodeling of the mitochondrial cristae following IL-1β stimulation, indicating mitochondrial health (Fig. 4B). IL-1β promoted an increase in the volume and circumference of individual mitochondria, as well as an increase in the number and length of mitochondrial branches, suggesting a trend toward fusion and increased networking of mitochondria (Fig. 4C). Upon further analysis of mitochondrial dynamics and fusion proteins, the expression of the mitochondrial fusion-related protein OPA1 was significantly increased in OLP-T cells under IL-1β stimulation (Fig. 4D and Supplementary Fig. S3A, B). The ratio of L-OPA1/S-OPA1 subtypes, which mediate fusion, was also significantly increased (Fig. 4E). L-OPA1 is the primary subtype involved in mitochondrial fusion, and its ratio to S-OPA1 may regulate the balance between mitochondrial fusion and fission [23]. Our observations of mitochondrial morphology supported this hypothesis. These findings suggest that IL-1β stimulates the expression of OPA1, particularly L-OPA1, promoting mitochondrial fusion and increasing NAD^+^ availability in OLP-T cells.

### OPA1 regulates energy metabolic reprogramming and activation of T cells in OLP

**Fig. 5:**
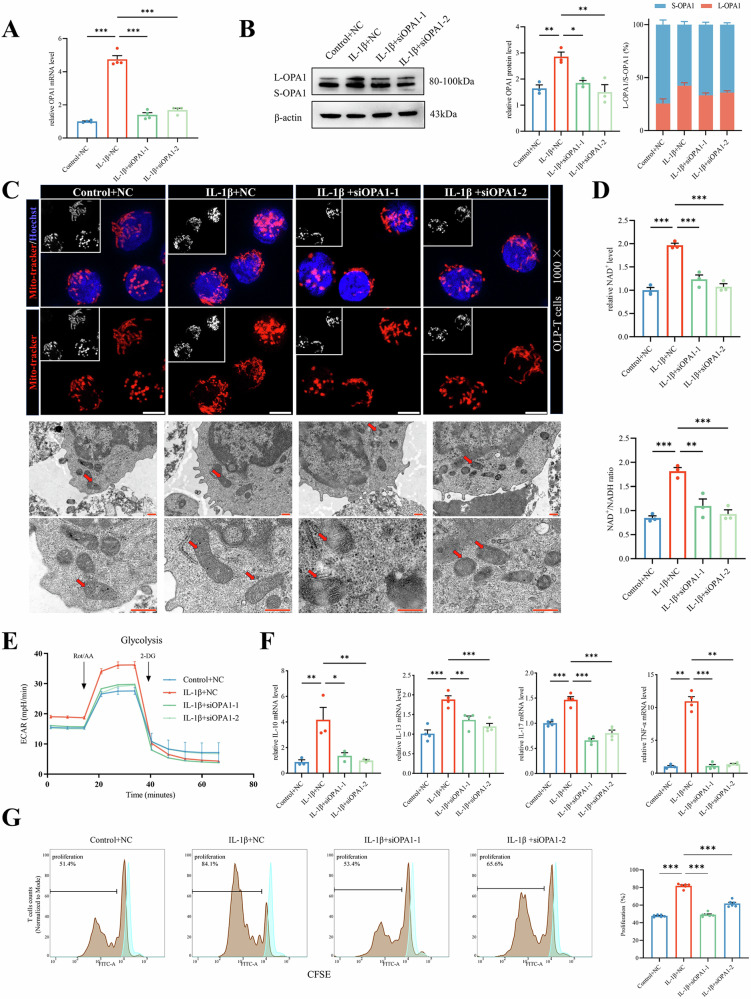
OPA1 regulates energy metabolic reprogramming and activation of T cells in OLP. **A**, **B** The mRNA and protein levels of OPA1 under indicated treatment in OLP-T cells with or without knockdown OPA1, OPA1 protein expression was analyzed by Image J. **C** Mitochondria in OLP-T cells with or without knockdown OPA1, captured by confocal microscopy (upper, Mitochondria labeled with Mitotracker, highlighted in red, and cell nuclei labeled with Hoechst, depicted in blue. bar: 5 μm), and transmission electron microscopy (lower, mitochondria indicated by red arrows, bar: 500 nm) after IL-1β treatment. **D** NAD^+^ content and NAD^+^/NADH ratio in OLP-T cells with or without knockdown OPA1, stimulated with exogenous IL-1β, detected by NAD^+^ Assay Kit. **E** Seahorse analysis of the glycolysis rate in OLP-T cells with or without knockdown OPA1 revealed variations in the ECAR over time. **F** The mRNA levels of inflammatory factors released after T-cell activation, under treatment with exogenous IL-1β. **G** CellTrace™ CFSE-labeled OLP-T cells with or without knockdown OPA1, analyzed using flow cytometry, under treatment with exogenous IL-1β, proliferation rates were quantitatively analyzed. The blue represents the primary peak without CD3/CD28 stimulation, the brown represents the proliferation peak under the indicated conditions. *P < 0.05. **P < 0.01. ***P < 0.001. OLP-T cells T cells from peripheral blood of patients with OLP, NC negative control siRNA.

After observing an increase in OPA1 expression, we investigated its role in mitochondrial fusion and T-cell activation. We identified two suitable siRNAs for subsequent experiments based on their effect on OPA1 expression (Supplementary Fig. S3C). In T cells with OPA1 knockdown, there was no significant increase in OPA1 expression, even when stimulated with IL-1β, and the L-OPA1/S-OPA1 ratio was lower than that in T cells treated with IL-1β alone (Fig. 5A, B). When treated with IL-1β, OPA1-knockdown T cells exhibited reduced mitochondrial area, circumference, and branching, indicating decreased fusion and networking (Fig. 5C and Supplementary Fig. S4A). OPA1-knockdown T cells did not show significant changes in NAD^+^ content or the NAD^+^/NADH ratio (Fig. 5D), and ECAR (Fig. 5E and Supplementary Fig. S4B) or the expression of glycolysis-related genes (Supplementary Fig. S4C). The ratio of ATP production to glycolysis was also inhibited, as shown in Supplementary Fig. S4D.

Analysis of proliferation and activation by RT-qPCR and flow cytometry indicated that IL-1β treatment did not significantly increase the mRNA levels of activation factors such as IL-10 in OPA1-knockdown T cells, and the leftward shift in the proliferation peak was inhibited as well (Fig. 5F, G). These results suggested that IL-1β affects the mitochondrial dynamics of OLP-T cells by regulating OPA1 expression, increasing NAD^+^ availability, and controlling metabolic reprogramming.

### IL-1β promotes OPA1 transcription via activating the NF-κB pathway, regulating energy metabolism reprogramming of T cells

**Fig. 6:**
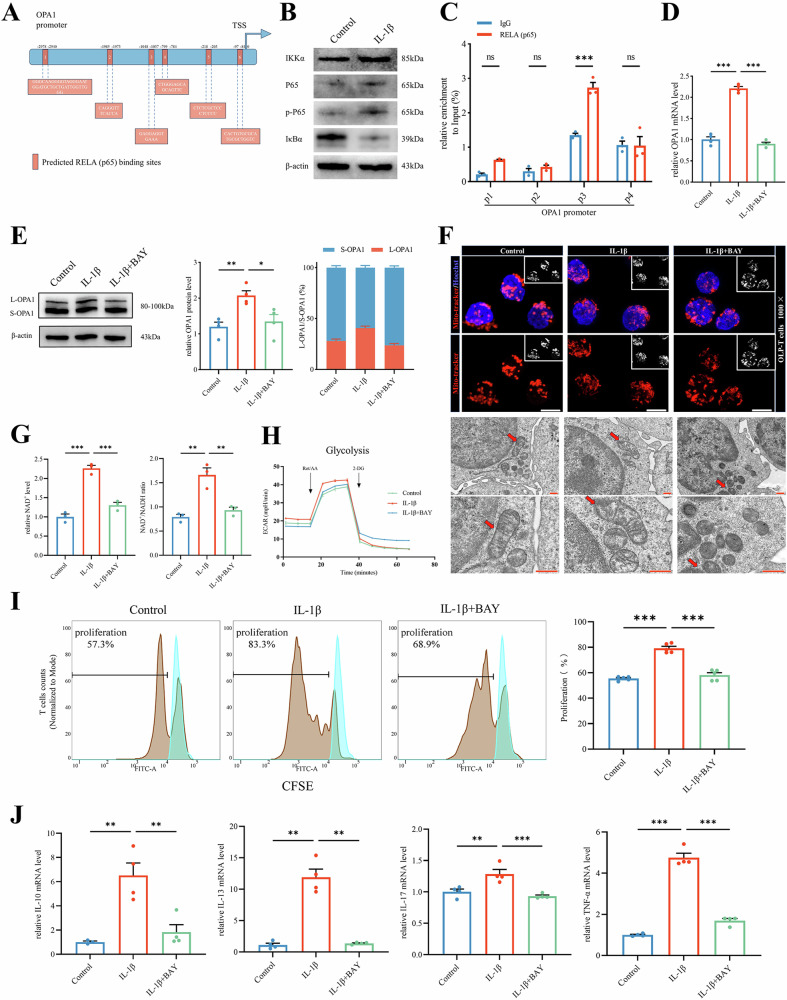
IL-1β promotes OPA1 transcription via activating the NF-κB pathway, regulating energy metabolism reprogramming of T cells. **A** Predicted binding sites of RELA (p65) to the OPA1 promoter region were identified using humanTFDB, Unibind, and HOCOMOCO. TSS denotes the transcription start site. **B** The protein levels of NF-κB pathway genes in OLP-T cells under IL-1β treatment. **C** ChIP-qPCR analysis revealed potential binding sites of RELA (p65) within the OPA1 promoter region. Four hypothesized RELA (p65) binding sites (P1-P4) within the OPA1 promoter were selected, and corresponding primers were designed and synthesized. ChIP-qPCR results indicated a high enrichment of RELA (p65) at the P3 region. **D**, **E** The mRNA and protein levels of OPA1 in OLP-T cells, under IL-1β treatment, with or without BAY, OPA1 protein expression was analyzed by Image J. **F** Mitochondria in OLP-T cells captured by confocal microscopy (upper, Mitochondria labeled with Mitotracker, highlighted in red, and cell nuclei labeled with Hoechst, depicted in blue. bar: 5 μm), and transmission electron microscopy (lower, mitochondria indicated by red arrows, bar: 500 nm) after IL-1β treatment, with or without BAY. **G** NAD^+^ content and NAD^+^/NADH ratio in OLP-T cells stimulated with exogenous IL-1β, with or without BAY. detected by NAD^+^ Assay Kit. **H** Seahorse analysis of the glycolysis rate in OLP-T cells revealed variations in the ECAR over time. **I** CellTrace™ CFSE-labeled OLP-T cells analyzed using flow cytometry, under the treatment with exogenous IL-1β, proliferation rates were quantitatively analyzed. The blue represents the primary peak without CD3/CD28 stimulation, the brown represents the proliferation peak under the indicated conditions. **J** The mRNA levels of inflammatory factors released after T-cell activation, under treatment with exogenous IL-1β, with or without BAY. *P < 0.05. **P < 0.01. ***P < 0.001. ChIP-qPCR chromatin immunoprecipitation followed by quantitative PCR, OLP-T cells T cells from peripheral blood of patients with OLP.

To investigate the relationship between IL-1β and transcriptional regulation of OPA1, we used the humanTFDB database for predictive analysis. Our findings revealed a strong correlation between the NF-κB subunit, RELA (p65), and the promoter region of OPA1 (Fig. 6A and Supplementary Table S3). Experimental investigations confirmed this relationship and showed that exogenous IL-1β degraded IκB-α, an NF-κB inhibitor, and increased the expression levels of the κB kinase inhibitor (IKK), ultimately resulting in enhanced phosphorylation of p65 in OLP-T cells (Fig. 6B and Supplementary Fig. S5A). These results indicate the activation of the NF-κB pathway in response to IL-1β stimulation. ChIP followed by quantitative PCR (ChIP-qPCR) was used to evaluate the four predicted p65 binding sites (P1-P4) on the OPA1 promoter. The analysis revealed significant enrichment of p65 in the P3 region, confirming the transcriptional modulation exerted by the NF-κB pathway upon IL-1β (Fig. 6C and Supplementary Fig. S5B).

Administration of BAY 11-7082, an NF-κB pathway inhibitor, resulted in a reduction in both the mRNA and protein levels of OPA1 in T cells treated with IL-1β. This also decreased the L-OPA1/S-OPA1 ratio (Fig. 6D, E), indicating the important role of NF-κB-mediated transcription in the regulation of metabolic dynamics. Phenotypic investigations were consistent with the molecular findings. The results showed that the disruption of NF-κB signaling through BAY 11-7082 impeded mitochondrial fusion and networking and reduced the ratio of NAD^+^/NADH (Fig. 6F, G and Supplementary Fig. S5C), as well as attenuated glycolytic activity in OLP-T cells under IL-1β stimulation, as evidenced by the reduced ECAR (Fig. 6H and Supplementary Fig. S5D). The protein levels of glycolysis-related genes, including GLUT1, HK2, and PKM2, were consistently decreased (Supplementary Fig. S5E). This led to the inhibition of T-cell proliferation and expression of activation factors, such as IL-10 and IL-13 (Fig. 6I, J). Therefore, these data suggest that IL-1β promotes OPA1 transcription by activating the NF-κB pathway, which further promotes mitochondrial fusion, enhances NAD^+^ availability and glycolysis, and ultimately contributes to OLP-T-cell proliferation and activation.

## Discussion

OLP is characterized by the infiltration of T cells into the submucosal layer and liquefactive degeneration of basal epithelial cells. Although basal cell death is thought to be due to apoptosis, it does not fully align with the apoptotic characteristics. Our research revealed, for the first time, that epithelial cells in the OLP undergo pyroptosis. This process involves the release of inflammatory factors from pyroptotic epithelial cells, which promotes the proliferation, and activation of T cells. Activated T cells then attack the epithelial cells, leading to their death. This study reveals a novel molecular mechanism underlying the development and progression of OLP from a new and insightful perspective.

Caspase-1 plays a crucial role in pyroptosis [24]. Activated caspase-1 cleaves the N-terminal fragment of GSDMD, which binds rapidly to liposomes and forms plasma membrane pores. This channel allows the release of inflammatory cytokines, such as IL-1β into the extracellular space [5, 25]. The OLP epithelium expresses high levels of pyroptosis-related cytokines IL-1β and IL-18, as well as inflammatory factors such as TNF-α, IL-12, and IL-6, which are several times higher than those found in the normal mucosa [26]. Patients with OLP exhibit elevated serum levels of IL-18 and polymorphisms in the IL-18 gene are linked to increased susceptibility to the condition [26]. Despite elevated IL-18 expression in lesions, no significant correlation exists between IL-18 levels and OLP severity in our study. However, the strong correlation between IL-1β levels and clinical severity of OLP suggests a significant association between pyroptosis and the progression of OLP.

CD8^+^ T cells require signals from the Th cells to exert specific cytotoxic effects. Naïve Th cells differentiate into Th1 or Th2 cells under the activation of different factors, secreting high levels of IL-2, IFN-γ, TNF-α, IL-4, IL-5, IL-10, and IL-13 [26]. Our study found that stimulating OLP-T cells with IL-1β led to increased expression of TNF-α, IL-10, IL-13, and IL-17. This suggests that IL-1β can promote the differentiation of OLP-T cells into Th1, Th2, and Th17 phenotypes. The relative dominance of Th1 and Th2 in OLP remains unresolved in previous studies, although both are recognized as having significant roles [27–29]. The use of neutralizing antibody or inhibitor against IL-1β can effectively suppress T-cell activation in patients with OLP, suggesting potential therapeutic targets for this condition.

This study found that IL-1β activates the NF-κB pathway in OLP-T cells. The NF-κB essential modulator (NEMO) binds to polyubiquitin chains on several upstream molecules, including IRAK1, forming a core IKK complex with IKK. Activated IKK phosphorylates IκBα, promoting its K48-linked polyubiquitination and subsequent proteasomal degradation. The degradation of IκB allows for the release and nuclear translocation of the NF-κB subunits p50 and p65 [30]. Our results suggest that IL-1β promotes the translocation of p65 to the nucleus for transcriptional regulation of downstream genes, as P65 serves as a key step in activating the NF-κB pathway [31]. Inhibiting OPA1 expression using an NF-κB pathway inhibitor suggests that the nuclear translocation of NF-κB-p65 promotes the transcriptional upregulation of OPA1. This shift favors the L-OPA1 isoform, augmenting the L-OPA1/S-OPA1 ratio and mediating mitochondrial fusion.

OPA1 is a crucial GTPase in mitochondrial dynamics that integrates into the IMM through its N-terminus. There are two splice forms of OPA1: the IMM-anchored long-form OPA1 (L-OPA1) and the soluble short-form OPA1 (S-OPA1). L-OPA1 is cleaved into S-OPA1 by OMA1 and YME1 Like 1 ATPase proteins, and the relative levels of L-OPA1 and S-OPA1 are crucial for mitochondrial fusion [32]. OPA1 plays a crucial role in IMM fusion and coordinates cristae integrity, mitochondrial DNA maintenance, bioenergetics, and assembly of respiratory chain supercomplexes. These functions directly affect mitochondrial cytochrome release and oxidative respiration efficiency [20]. Changes in mitochondrial morphology and metabolic reprogramming are important drivers of normal T-cell maturation and function [23]. When stimulated with IL-1β, T cells with knockdown OPA1 exhibited reduced mitochondrial fusion and proliferative activation. This study emphasized the regulatory role of the NF-κB pathway in OPA1-mediated mitochondrial fusion in OLP-T cells.

Glycolysis and mitochondrial respiration depend on NAD^+^ for cellular metabolism. The Warburg effect is a representative adaptation of NAD^+^ regeneration from NADH in metabolically active cells, suggesting that cells primarily rely on glycolysis for NAD^+^ regeneration in the absence of OXPHOS [33]. NAD^+^ regeneration occurs through two cyclic pathways. The first is the main mechanism of glycolysis in the absence of OXPHOS, which converts pyruvate, a key mitochondrial substrate, into lactate, while transforming NADH back into NAD^+^. Mitochondrial NADH shuttles, including glycerol-3-phosphate (G3P) and malate/aspartate shuttles (MAS), as well as the activity of respiratory chain complex I in the IMM, provide an alternative pathway for NAD^+^ regeneration [34, 35]. This study showed that OPA1-mediated mitochondrial fusion in T cells regulates the NAD^+^/NADH ratio, which is essential for the continuous function of aerobic glycolysis [19]. Subcellular distribution of NAD^+^ between the cytoplasm and mitochondria can also affect the characteristics of OXPHOS and glycolytic metabolism [36], indicating the involvement of complex mechanisms that require further investigation.

During the activation and proliferation of OLP-T cells, both OXPHOS and glycolysis were enhanced. Previous studies have suggested that mitochondrial fission in T cells can reduce the activity of the electron transport chain, leading to the inhibition of OXPHOS and enhancement of glycolysis [12]. This indicates a competitive relationship between the two energy supply pathways. Some researchers argue that enhanced aerobic glycolysis does not necessarily inhibit OXPHOS. They suggested that aerobic glycolysis might be facilitated by increased respiration, leading to metabolic shifts. This implies that more glucose is involved in glycolysis than in oxidation [17, 37]. Bezafibrate can enhance OXPHOS and glycolysis in cytotoxic T lymphocytes, promoting the proliferation and functionality of immature T cells [20]. Our finding also supports the notion that activated T cells require increased glycolysis for energy supply [10].

Several key glycolytic enzymes are involved in T-cell proliferation and activation following IL-1β stimulation. GLUT1 can translocate to the cell surface in response to activation of the phosphatidylinositol 3-kinase (PI3K)-Akt pathway, which enhances glucose uptake [38]. The activities of HK2, PFKFB3, and PKM2 have been reported to be highly correlated with glucose uptake, clonal expansion, and T-cell proliferation [39–41]. PFKP and PFKM, platelet and muscle isoforms of PFK, play roles in the metabolic adaptation of T cells [42, 43]. In our study, co-treatment of T cells with the glycolytic inhibitor 2-DG and IL-1β did not result in a significant increase in the expression of glycolysis-related genes. However, no significant changes were observed in the expression levels of LDHA. Studies have found that the expression level of LDHA in neuroblastomas is not correlated with glycolytic activity [44]. In addition, inhibition of LDHA results in increased glycolysis in MDA-MB-231 breast cancer cells [45]. Therefore, the relationship between LDHA expression and glycolysis in OLP-T cells might involve more complex mechanisms.

A major limitation of this study is the absence of effective animal models for OLP, restricting deeper mechanistic insights and the exploration of treatments in vivo. Consequently, our findings are based on in vitro and clinical analyses, highlighting the necessity for developing appropriate animal models in future research.

In conclusion, IL-1β released during keratinocyte pyroptosis plays a crucial role in promoting the proliferation and activation of OLP-T cells. IL-1β activates the NF-κB pathway, promotes OPA1-mediated mitochondrial fusion of T cells, and facilitates the availability of NAD^+^, thus participating in energy metabolism reprogramming of T cells to ensure the supply of ATP needed for proliferation and activation, thus contributing to the occurrence of OLP. These findings provide new insights into the mechanisms and therapeutic strategies of OLP.

## Materials and methods

### Immunohistochemistry (IHC) analyses and histopathology

The acquisition and execution of the experimental materials were approved by the Committee on Human Research of the School and Hospital of Stomatology at Sun Yat-sen University (KQEC-2024-05-02). The study sample included 19 male and 11 female patients. Written informed consent was obtained from all the patients.

Oral mucosal tissues were collected from 11 healthy individuals and 30 patients diagnosed with OLP, of whom 15 were non-erosive and erosive. The tissue sections were incubated with the indicated antibodies overnight at 4 °C. Subsequently, HRP peroxidase-conjugated anti-rabbit or anti-mouse secondary antibodies were added and incubated for 1 h at 37 °C. Images were acquired at 200× and 400× magnification using a Leica Aperio AT2 microscope (#8482, Leica, Hesse, Germany). The staining results were evaluated semi-quantitatively based on the ratio of the staining intensity to the proportion of positive cells.

### REU scores of patients with OLP

The diagnosis of OLP was based on the presence of bilateral symmetric white striations or papules with or without erythema, erosions, and/or ulceration (ulceration was defined as a yellow-white fibrin membrane), and a biopsy was read as OLP. The clinical signs of OLP were measured using a semiquantitative REU scoring system (Supplementary Table S2).

Oral cavity was divided into 10 sites in the REU scoring system: right buccal mucosa, left buccal mucosa, tongue dorsum, tongue ventrum, maxillary gingiva, mandibular gingiva, floor of mouth, hard palatal mucosa, soft palate and tonsil, and labial mucosa (upper and lower together). The severity of the lesion in each site was scored according to the following: presence of reticular/hyperkeratotic/white papular (R) lesions (0 = none, 1 = presence), presence of each erosive/erythematous (E) lesions and/or ulcerative (U) lesions (0 = none, 1 = lesions smaller than 1 cm^2^, 2 = lesions from 1 to 3 cm^2^, 3 = lesions larger than 3 cm^2^). Each REU score was totaled from all 10 areas, and the total weighted score was a summation of reticulation score, erythematous score (weighted 1.5), and ulcerative score (weighted 2.0) [46].

### Isolation and culture of oral keratinocytes and CD3^+^ T cells

Mucosal samples were digested using 0.25% Dispase II (4942078001-1, Roche, Basel, Switzerland) at 4 °C for 12 h. Epithelial samples were then oscillated and digested at 37 °C for 5 min. Digestion was terminated by the addition of calf serum. Keratinocytes were resuspended in DK-SFM medium containing penicillin (100 U/mL) and streptomycin (100 μg/mL).

Peripheral blood mononuclear cells (PBMCs) were isolated from blood samples of healthy individuals and patients with OLP by density gradient centrifugation. CD3^+^ T cells were isolated from PBMCs using the EasySep™ Human CD3 Positive Selection Kit II (17851, Stemcell, Vancouver, Canada), according to the manufacturer’s instructions. The isolated cells were resuspended and cultured in a T-cell medium.

The co-culture system was achieved through indirect co-culture, in which half of the culture supernatant from keratinocytes was mixed with half of the T-cell culture medium.

### RT-qPCR and western blotting

RT-qPCR was conducted using ChamQ Universal SYBR qPCR Master Mix (Q711-02, Vazyme) and QuantStudio™ 7 Pro (Thermo Fisher Scientific, Massachusetts, USA). The mRNA expression levels were normalized to the expression of housekeeping genes (β-actin or HPRT1).

For western blot analysis, protein concentrations were determined using a BCA kit (CW0014s, Cwbio, Jiangsu, China). Protein extracts were separated using SDS-PAGE and transferred to the PVDF membranes (ISEQ00010, Merck Millipore, New Jersey, USA). Membranes were visualized using a chemiluminescence imaging system (Bio-Rad, California, USA). The results were analyzed semi-quantitatively using Image Lab and Image J software.

### Cell proliferation experiments and flow cytometry

For the cell proliferation experiments, CD3^+^ T cells were labeled with CellTrace CFSE (Invitrogen, California, USA). The samples were acquired using a BD LSRFortessa flow cytometer (Becton, Dickinson and Company) and analyzed using the FlowJo software (TreeStar).

### Imaging analysis of mitochondrial structure

MitoTracker (M7512, Thermo Fisher Scientific) and Hoechst 33342 (C1025, Beyotime, Jiangsu, China) staining was performed according to the manufacturer’s instructions. Images were acquired using an Olympus Confocal Microscope FV3000 with a 100× objective lens. Confocal images were analyzed using the mitochondrial analyzer in Image J software.

For transmission electron microscopy (TEM), cells were fixed in glutamate fixative (2.5% glutaraldehyde) for electron microscopy. The resin blocks were sliced to a thickness of 60–80 nm using an ultramicrotome. The tissues were then affixed to 150-mesh copper grids coated with a formvar film. The copper grids were examined using a Transmission Electron Microscope (HT7800/HT7700; HITACHI).

### Metabolic assays

A Seahorse XFe96 Extracellular Flux Analyzer was used to perform ECAR and OCR measurements. The Seahorse XF ATP rate or Glycolytic Rate Assay Kit (Agilent Technologies, California, USA) was used, according to the manufacturer’s instructions.

### Cell transfection

RiboBio (Guangzhou, China) chemically synthesized siRNAs. A negative control siRNA (siRNA-NC) with random sequences targeting unknown mammalian genes was used. Lipofectamine 3000 (L3000075; Thermo Fisher Scientific) was used for transfection.

### Bioinformatics

The Eukaryotic Promoter Database (EPD) was used to search for human gene promoter sequences. The PathCards database of human biological pathways was used to search for pathways associated with IL-1β expression. Predictive analysis of the binding sites between RELA (p65) and the promoter region of OPA1 was conducted using humanTFDB, Unibind, and HOCOMOCO databases.

### Chromatin immunoprecipitation assay (ChIP)

The ChIP assay was performed using a ChIP Assay Kit (Beyotime, China) following the manufacturer’s instructions. Briefly, T cells were cross-linked with a 37% formaldehyde solution for 10 min at room temperature and quenched with 125 mM glycine. Ultrasonication was used to obtain DNA fragments ranging in size from 200 to 500 bp. The lysate was immunoprecipitated with anti-RELA (p65) or IgG antibodies. The immunoprecipitated DNAs were analyzed using RT-qPCR.

### Statistical analysis

Statistical analyses were performed using SPSS (version 24.0; USA) and Microsoft Excel. Statistical significance was set at *P* < 0.05. Data are presented as mean ± SEM (standard error of the mean).

More detailed materials and methods are described in the Supplementary file.

## Supplementary information

supplementary materials

original western blots

## Data Availability

The data from the current study are available upon reasonable request.
