# Supplementary material for: Pyroptosis of oral keratinocyte contributes to energy metabolic reprogramming of T cells in oral lichen planus via OPA1-mediated mitochondrial fusion
Source: Cell Death Discov. 2024 Sep 17;10:408. doi: 10.1038/s41420-024-02174-1 (PMC11408637; doi:10.1038/s41420-024-02174-1)
Supplement: Supplementary file 1 — supplementary materials [file 41420_2024_2174_MOESM1_ESM.pdf]

supplementary figures 1-5

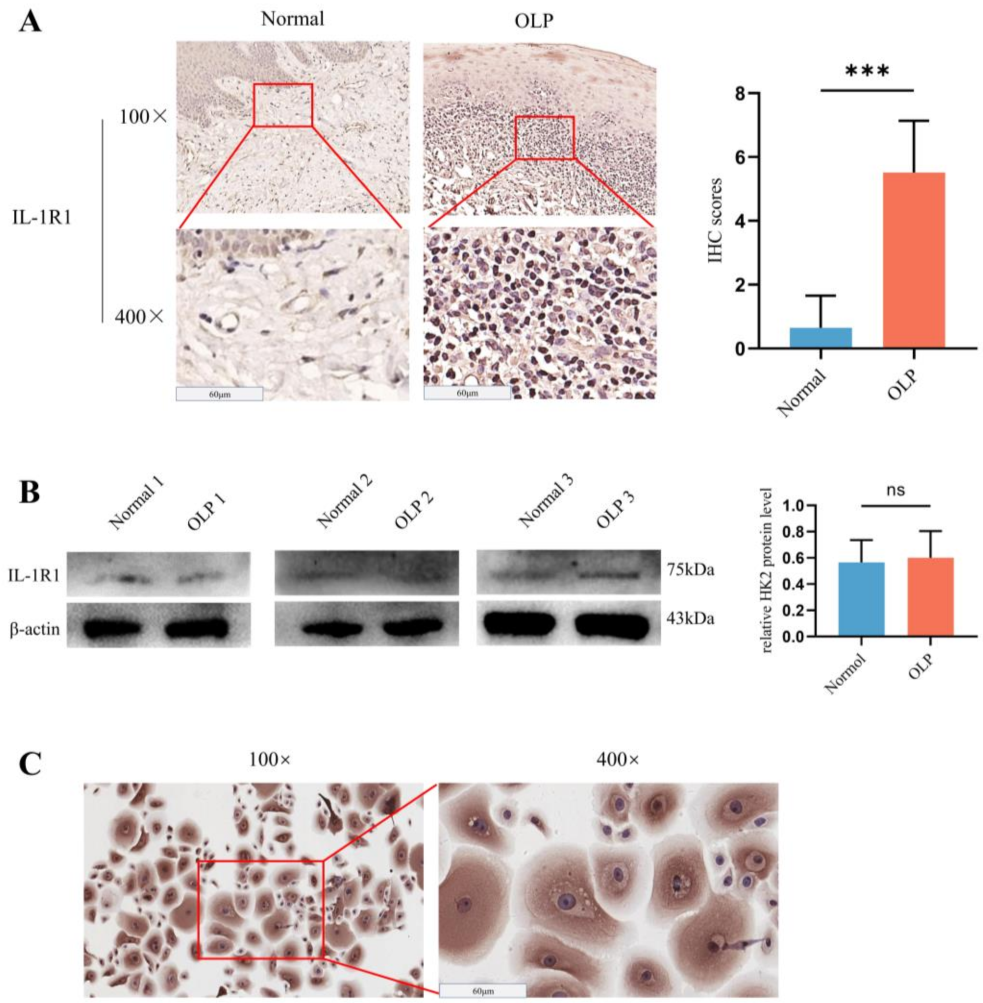

Fig. S1. T cells in OLP patients express receptors for IL-1β.

(a)IHC assays for IL-R1 proteins in T-cells of mucosal tissues. Sample sizes: Normal: n=10, OLP: n=15, IHC semi-quantitative analysis of IL-1R1 expression of lymphocyte in mucosal epithelial tissues.

(b)The protein levels of IL-1R1 genes in OLP-T cells under indicated treatment, semi-quantitative analysis using Image J.

(c) The AE1/AE3 keratin immunocytochemical staining of primary oral keratinocytes.

IL-R1, receptors 1 for IL-1β; OLP, oral lichen planus.

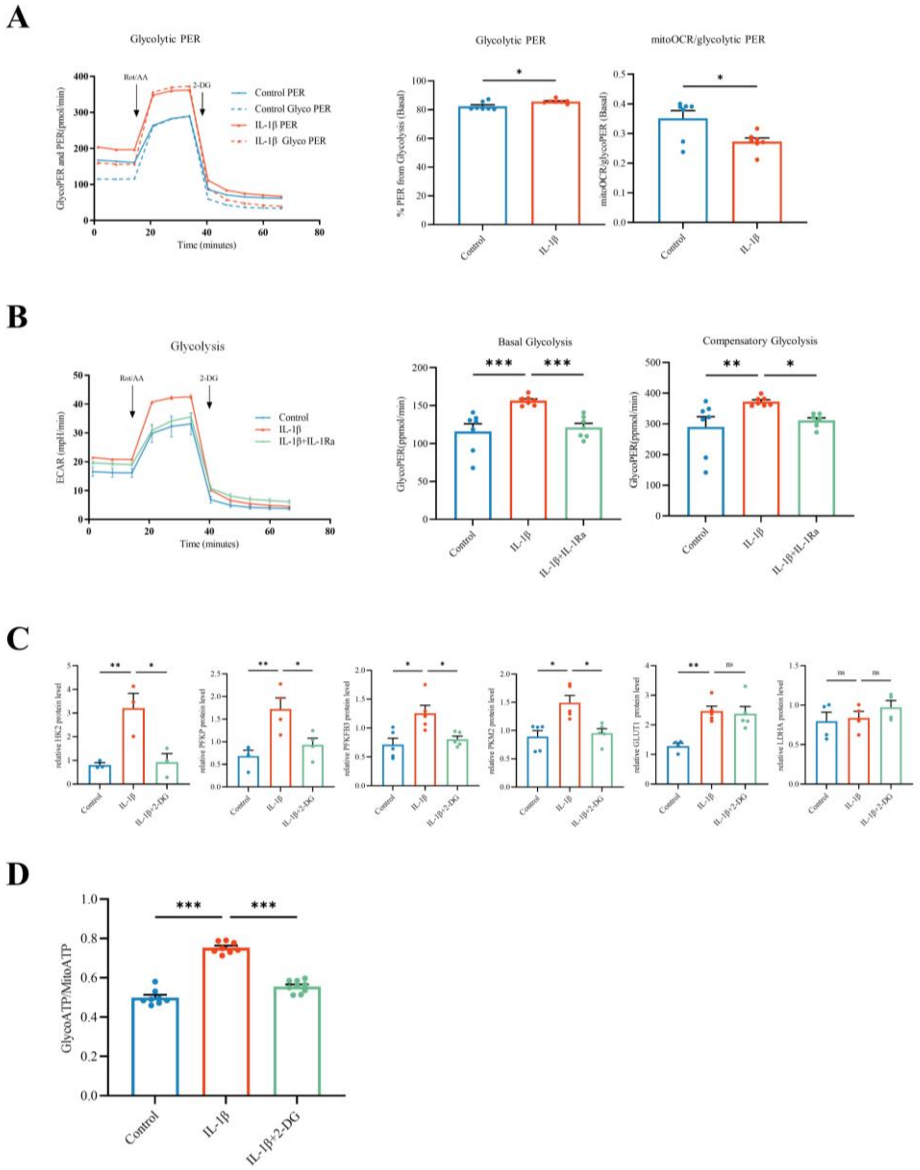

Fig. S2. IL-1β regulates the metabolic reprogramming of T cells.

(a) Seahorse ® analysis of the glycolysis rate in OLP-T cells showed variations in the total PER and glycolytic PER over time, and ECAR over time (b), quantification by Agilent Seahorse Wave.

(c) The protein levels of glycolysis-related genes in OLP-T cells under indicated treatment, semi-quantitative analysis using Image J.

(d) Seahorse® analysis of ATP production rate and source changes in OLP-T cells, quantification by Agilent Seahorse Wave.

* $P < 0.05$. **$P < 0.01$. ***$P < 0.001$. OLP-T cells, T cells from peripheral blood of patients with OLP; 2-DG, 2-deoxy-D-glucose; PER, Proton Efflux Rate; OCR, Oxygen Consumption Rate; ECAR, Extracellular Acidification Rate.

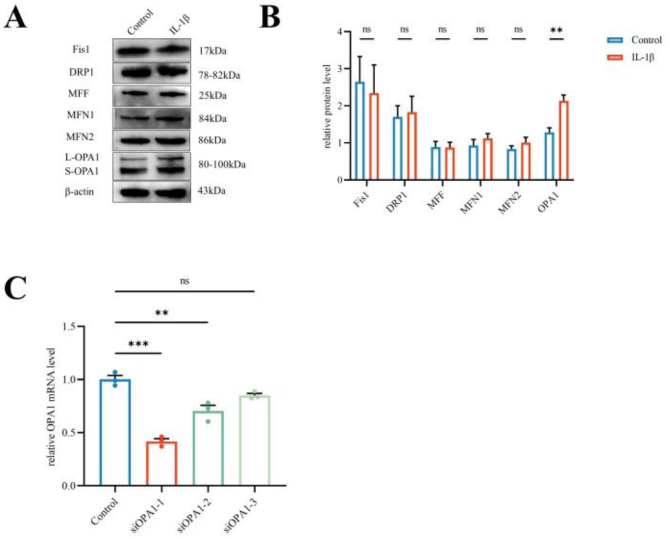

Fig. S3. IL-1β upregulates OPA1 expression in OLP-T cells, siRNA knockdown of OPA1.

(a-b) The protein levels of mitochondrial dynamic-related genes in OLP-T cells under indicated treatment, semi-quantitative analysis using Image J.

(c) The mRNA levels of OPA1 in OLP-T cells under 3 different siRNAs to knockdown OPA1.

* $P < 0.05$. **$P < 0.01$. ***$P < 0.001$. OLP-T cells, T cells from peripheral blood of patients with OLP.

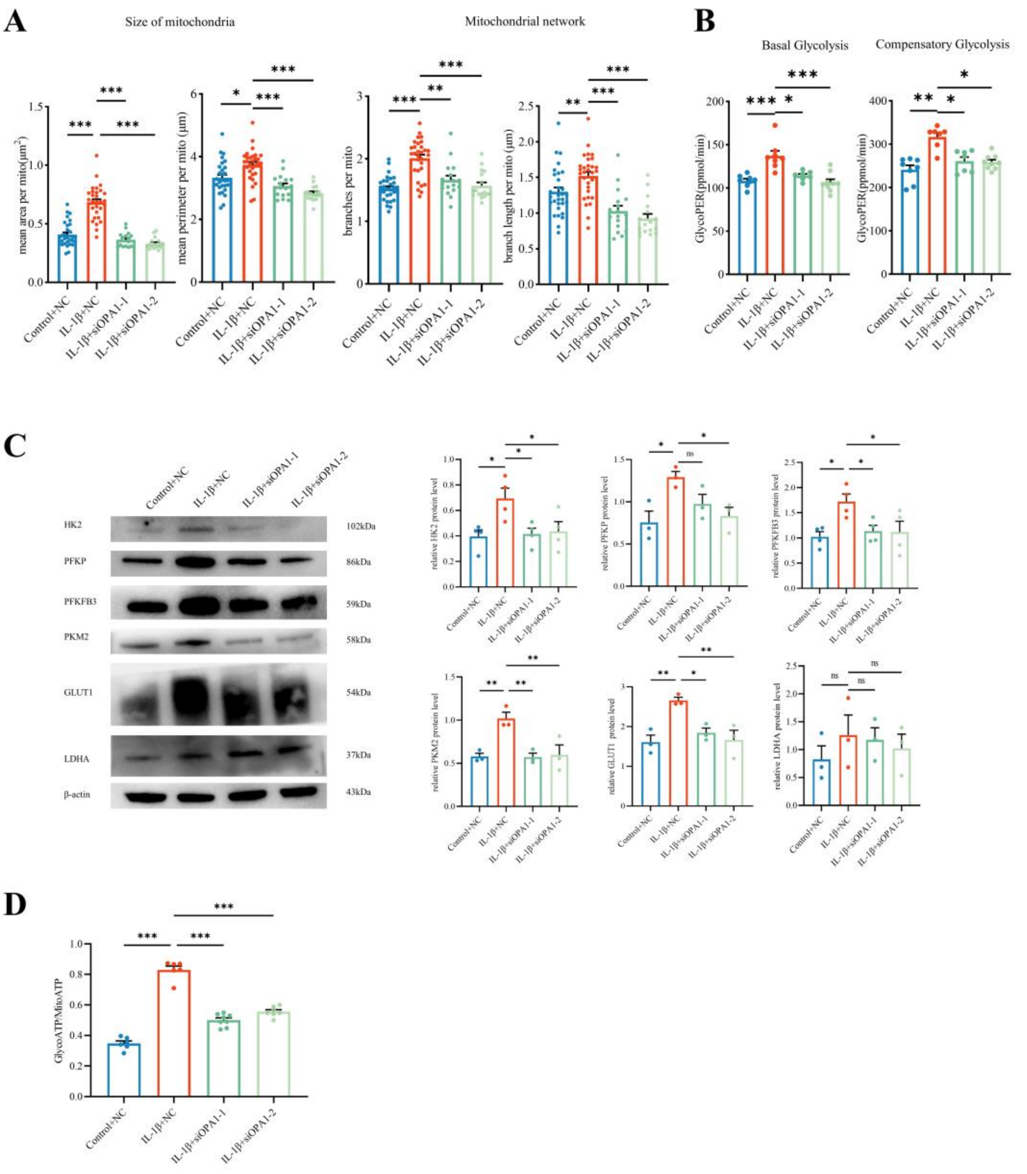

Fig. S4. IL-1β promotes mitochondrial fusion and glycolytic activity in OLP-T cells.

(a) Mitochondria in OLP-T cells with or without knock down OPA1, labelled with Mitotracker captured by confocal microscopy under indicated treatment, confocal images were analysed using Image J to assess the size and network structure of mitochondria.

(b) Seahorse® analysis of the glycolysis rate in OLP-T cells with or without knock down OPA1, under indicated treatment, quantification by Agilent Seahorse Wave.

(c) The glycolysis-related protein levels in OLP-T cells with or without knock down OPA1, under indicated treatment (upper), semi-quantitative analysis using Image J (lower).

(d) Seahorse® analysis of ATP production rate and source changes in OLP-T cells with or without knock down OPA1, quantification by Agilent Seahorse Wave.

* $P < 0.05$. **$P < 0.01$. ***$P < 0.001$. OLP-T cells, T cells from peripheral blood of patients with OLP; 2-DG, 2-deoxy-D-glucose; PER, Proton Efflux Rate; OCR, Oxygen Consumption Rate; ECAR, Extracellular Acidification Rate; NC, negative control siRNA.

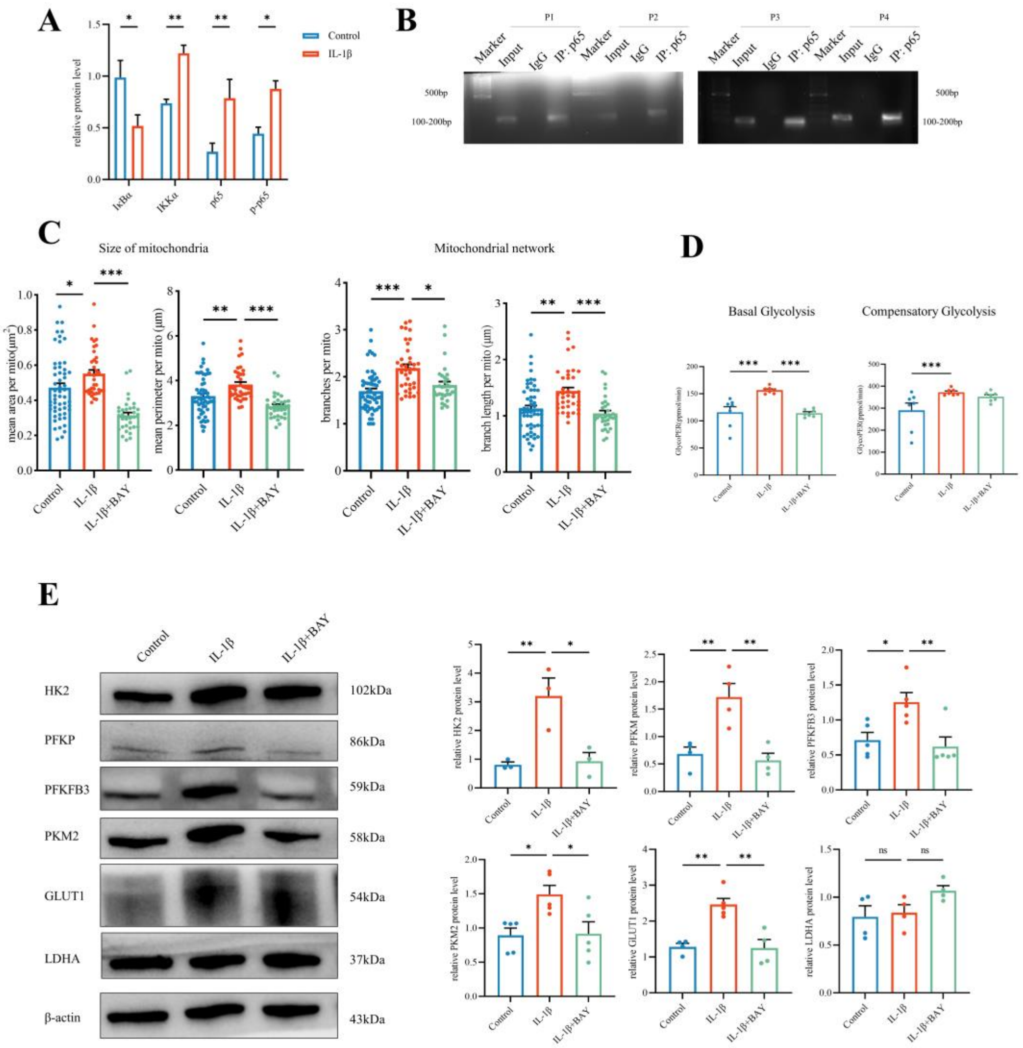

Fig. S5. IL-1β promotes mitochondrial fusion and glycolytic activity in OLP-T cells through the NF-kB pathway.

(a) The protein levels of NF-κB pathway-related genes in OLP-T cells under indicated treatment, semi-quantitative analysis using Image J.

(b) Agarose gel electrophoresis after ChIP-qPCR analysis for revealing potential binding sites of RELA (p65) within the OPA1 promoter region.

(c) Mitochondria in OLP-T cells labelled with Mitotracker captured by confocal microscopy under indicated treatment, confocal images were analysed using Image J to assess the size and network structure of mitochondria.

(d) Seahorse® analysis of the glycolysis rate in OLP-T cells under indicated treatment, quantification by Agilent Seahorse Wave.

(e) The protein levels of glycolysis-related genes in OLP-T cells under indicated treatment, semi-quantitative analysis using Image J.

* $P < 0.05$. **$P < 0.01$. ***$P < 0.001$. OLP-T cells, T cells from peripheral blood of patients with OLP; 2-DG, 2-deoxy-D-glucose; PER, Proton Efflux Rate; OCR, Oxygen Consumption Rate; ECAR, Extracellular Acidification Rate.

supplementary file: material and methods

Material and methods

Samples, Immunohistochemistry (IHC) analyses and histopathology

The acquisition and execution of the experimental materials were approved by the Committee on Human Research of the School and Hospital of Stomatology at Sun Yat-sen University (KQEC-2024-05-02). The study sample included 19 male and 11 female patients. Written informed consent was obtained from all the patients.

Oral mucosal tissues were collected from 11 healthy individuals and 30 patients diagnosed with OLP, of whom 15 were non-erosive and erosive. Tissues were fixed and embedded in paraffin. 5-μm sections were stained with haematoxylin and eosin. The formalin-fixed, paraffin-embedded oral mucosal tissues were prepared, sectioned at 4 micrometers and analyzed for the presence of indicated antigen on the basis of standard immunohistochemistry protocols. The tissue sections were incubated with the indicated antibodies overnight at 4 °C. Subsequently, HRP peroxidase-conjugated anti-rabbit or anti-mouse secondary antibodies were added and incubated for 1h at 37 °C. Images were acquired at 200× and 400× magnification using a Leica Aperio AT2 microscope (#8482, Leica, Germany). The staining results were evaluated semi-quantitatively based on the ratio of the staining intensity to the proportion of positive cells.

The positive signal appeared as brownish-yellow staining in the cell nuclear/cytoplasm. All slides were evaluated by two senior oral pathologists independently. The staining results were semi-quantitatively evaluated, based on the ratio of the staining intensity and proportion of positive cells. The intensity score was defined as 0, negative; 1, weak; 2, moderate; or 3, strong, brown. The proportion score was defined as 0, negative; 1, $<$ 10%; 2, 11–50%; 3, 51–80%; or 4, $>$ 80% positive cells. The immunoreactive score was calculated as the intensity score × proportion score [1].

The working dilutions of antibodies in IHC: Anti-IL-1β (1:1000), anti-IL1 Receptor I/IL-1R-1 (1:500), anti-cleaved N-terminal GSDMD (1:500), anti-Caspase 1(1:500), anti-Cleaved-Caspase 1 p20 (1:500) and anti-IL-18(1:1000).

The inclusion of OLP cases was based on the diagnostic criteria of the World Health Organization, and the diagnosis of OLP was confirmed through pathological examination. Patients with any of the following conditions were excluded from consideration: (1) pregnant or breastfeeding; (2) individuals with drug allergies, including those allergic to anesthetics; (3) patients for whom a biopsy was not feasible due to various factors; (4) individuals with other systemic or autoimmune diseases; (5) heavy smokers and drinkers; (6) those with other diseases or infections related to the oral mucosa; (7) patients who had taken antibiotics or immunomodulatory drugs within the last three months.

Disease scores of patients with OLP

OLP was diagnosed as follows: (1) presence of bilateral, symmetric white striations or papules, with or without erythema/erosions and/or ulceration (ulceration was defined as yellow-white fibrin membrane), and (2) a biopsy read as OLP. Clinical signs of OLP were measured using a semiquantitative Reticulation/keratosis, erythema, and ulceration (REU) scoring system (Table. S1). In this scoring system, the oral cavity was divided into 10 sites: right buccal mucosa, left buccal mucosa, tongue dorsum, tongue ventrum, maxillary gingiva, mandibular gingiva, floor of mouth, hard palatal mucosa, soft palate and tonsil, and labial mucosa (upper and lower together). The severity of the lesion in each site was scored according to the following: presence of reticular/hyperkeratotic/white papular (R) lesions (0 = none, 1 = presence), presence of each erosive/erythematous (E) lesions and/or ulcerative (U) lesions (0 = none, 1 = lesions smaller than 1 cm^2, 2 = lesions from 1 to 3 cm^2, 3 = lesions larger than 3 $cm^{2)}$. Each REU score

was totaled from all 10 areas and the total weighted score was a summation of reticulation score, erythematous score (weighted 1.5), and ulcerative score (weighted 2.0) [2].

Isolation of oral keratinocytes and CD3^{+}T cells

Biopsies of oral mucosa were collected from patients were diagnosed was obtained under sterile conditions and divided into two even parts for pathological examination or cell culture. Normal oral mucosal tissue is derived from normal oral mucosal tissue obtained from orthognathic surgery, periodontal surgery, implant surgery or mandibular third molar extraction at the Oral and Maxillofacial Surgery Department. Mucosal samples were placed in DK-SFM medium (10744019, Thermo fisher Scientific) containing penicillin (100 U/mL) and streptomycin (100 μg/mL) (15140122, Gibco USA). Mucosal samples were transported to the laboratory on 4℃ and, under sterile conditions, rinsed with phosphate-buffered saline (PBS) containing 2% antibiotics to remove blood. The samples were digested with 0.25% Dispase II (4942078001-1, Roche) at 4°C for 12 hours or in a 37°C water bath for 2 hours to separate the epithelial and connective tissues. After digestion, epithelial and subepithelial layers were gently peeled apart under a microscope using microscopic surgical tweezers. The isolated epithelium was rinsed three times with PBS, followed by microsurgical cutting of the epithelial tissues while maintaining tissue moisture. Subsequently, the tissues were oscillated and digested with 0.025% trypsin without EDTA at 37°C for 5 minutes. Digestion was terminated with serum-containing medium, and after centrifugation, the supernatant was discarded[3]. DK-SFM medium serves as an inhibitor of fibroblast growth, making it suitable for the extraction of epithelial cells that are prone to fibroblast contamination. Peripheral blood mononuclear cells (PBMCs) were isolated from blood samples of healthy individuals and patients with OLP using human peripheral blood lymphocyte isolation fluid (LTS1077, TBD) by

density gradient centrifugation. $CD3^+$ T cells were isolated from PBMCs using the EasySep™ Human CD3 Positive Selection Kit II (17851, Stemcell), according to the manufacturer's instructions. In brief, collect the PBMCs obtained in the previous step and resuspend in PBS, move to a flow tube, mix well, centrifuge at 2000 rpm for 5 min, and discard the supernatant. Resuspend, add CD3 antibody and incubate at 37°C for 5 min, then add magnetic beads and mix gently, incubate at 37°C for 10 min, flicking the flow tube every 3 min. Add 2 ml PBS mixed and place the flow tube into the magnetic rack (18000, Stemcell) for 5 min. The lymphocytes will be attracted to one side wall by the magnetic rack, discard the supernatant, resuspend and add CD3 antibody and magnetic beads and incubate at 37°C for a total of 3 times. The isolated cells were resuspended and cultured with T cell medium.

Culture of Cells and drug treatments

The keratinocytes were resuspended in DK-SFM medium containing penicillin (100 U/mL) and streptomycin (100 μg/mL). The cultures were incubated in an incubator (Thermo fisher Scientific, USA) with 5 % CO_2 and saturated humidity at 37 °C. Medium was replaced every 2 to 3 days.

For in vitro T cell activation, 0.5-1.0× 10^6 $CD3^+$ T cells were cultured on a 24-well plate pre-coated with 5 μg/ml anti-CD3 (300438, Biolegend) in T cell medium [RPMI/1640, 10% fetal bovine serum, 2 mM glutamine, 1 mM sodium pyruvate, 10 mM HEPES, 55 μM β-mercaptoethanol, 100 U/ml penicillin, 100 mg/ml streptomycin] supplemented with 2 μg/ml anti-CD28 (302934, Biolegend). The cultures were incubated in an incubator (Thermo fisher Scientific, USA) with 5 % CO_2 and saturated humidity at 37 °C. Medium was replaced half every 2 to 3 days. Where indicated, recombinant human IL-1β (10ng/mL, 200-01B-10, PeproTech), IL-1β neutralizing antibodies (2μg/mL 14-7018-81, eBioscience), IL-1Ra (200 ng/ml, HY-P7029, MCE) or 2-DG (2.5mM, S4701, Selleck) were added at the start of the cultures. Where

indicated, BAY 11-7082 (5μM, S2913, Selleck) were added at the 48h before collecting samples.

The conditional co-culture system is achieved through indirect co-culture, in which half of the culture supernatant from keratinocytes was mixed with half of the culture medium from T cell.

Antibodies

Anti-OPA1(612606) were obtained from BD Transduction Laboratories™. Anti-IL-1β(3A6) (#12242S), anti-Drp1(D8H5) (5391T), anti-LDHA(C28H7) (3558S), anti-MFF (E5W4M) (84580T), anti NF-κB p65 (D14E12) (8242T), anti Phospho-NF-κB p65 (Ser536) (93H1) (3033T), anti-rabbit and Anti-mouse IgG HRP-linked Antibody (7074S and 7076s) were obtained from Cell Signaling. Anti-IL1 Receptor I/IL-1R-1 (ab106278) and Anti-PFKP (ab119796) were obtained from Abcam. Anti-PFKFB3 (HY-P80275) and anti-Glucose Transporter (GLUT1) (HY-P80494) were obtained from MCE.Anti-PKM2 (15822-1-AP), anti-Hexokinase2 (HK2) (22029-1-AP) and anti-FIS1 (10956-1-AP) were obtained from Proteintech. Anti-Phospho-IκB alpha (340776) and anti-IκB alpha (383322) were obtained from Zen BioScience. Anti-IKK alpha/beta (AF6014), anti-Caspase 1(AF5418) and anti-Cleaved-Caspase 1 p20 (Asp296) (AF4005), were obtained from Affinity Biosciences. And other antibodies include , anti-GSDMDC1 (NBP2-33422, Novus), anti-MFN1 (BM4882, Boster), anti-MFN2 (AF7473, Beyotime), anti-beta Actin (EM31011-02, EMAR) and anti-IL-18(06-1115, Merck).

RT-qPCR and western blotting

RNA isolations were done by using the RNA Quick Purification kit (RN001, ESscience) and single-strand cDNA was synthesized using the HiScript III RT SuperMix for qPCR (R323-01, Vazyme). RT-PCR was performed with ChamQ Universal SYBR qPCR Master Mix (Q711-02, Vazyme) using

QuantStudio™ 7 Pro (Thermo Fisher Scientific, USA). The mRNA expression levels were normalized to the expression of housekeeping genes (β-actin or HPRT1). The primers used were:

Gene	5'to3'	3'to5'
MFN1	GGCCACATGTAGTTTATGTTTCCT	TGCACCTGCTGTAAAAAGGC
MFN2	GGAAGGTGAAGCGCAATGTC	TGCATTCACCTCAGCCATGT
OPA1	GCCACTTCCTGGGTCATTCC	CAGACCTCACAGGCCACAG
ACTB	CATGTACGTTGCTATCCAGGC	CTCCTTAATGTCACGCACGAT
HPRT1	CCTGGCGTCGTGATTAGTGAT	AGACGTTCAGTCCTGTCCATAA
Fis1	GTCCAAGAGCACGCAGTTTG	ATGCCTTTACGGATGTCATCATT
Drp1	CTGCCTCAAATCGTCGTAGTG	GAGGTCTCCGGGTGACAATTC
MFF	ACTGAAGGCATTAGTCAGCGA	TCCTGCTACAACAATCCTCTCC
GLUT1	ATTGGCTCCGGTATCGTCAAC	GCTCAGATAGGACATCCAGGGTA
HK2	TGCCACCAGACTAAACTAGACG	CCCGTGCCCACAATGAGAC
PFKM	AGCTGCCTACAACCTGGTGA	TCCACTCAGAACGGAAGGTGT
PKM2	ATGTCGAAGCCCCATAGTGAA	TGGGTGGTGAATCAATGTCCA
LDHA	ATGGCAACTCTAAAGGATCAGC	CCAACCCCAACAACTGTAATCT
PFKFB3	ATTGCGGTTTTCGATGCCAC	GCCACAACTGTAGGGTCGT
IL-4	CGGCAACTTTGTCCACGGA	TCTGTTACGGTCAACTCGGTG
IL-10	GACTTTAAGGGTTACCTGGGTTG	TCACATGCGCCTTGATGTCTG
IL-13	GAGGATGCTGAGCGGATTCTG	CACCTCGATTTTGGTGTCTCG
IL-17	AGATTACTACAACCGATCCACCT	GGGGACAGAGTTCATGTGGTA

TNF-α	GAGGCCAAGCCCTGGTATG	CGGGCCGATTGATCTCAGC

For western blot analysis, cells were washed with ice cold PBS and lysed in 1x lysis buffer (LB001, ESscience) supplemented with 1% phosphatase inhibitors and protease inhibitors. Samples were centrifuged at 12,000g for 20min at 4°C. Protein concentrations were determined with a BCA kit (CW0014s, Cwbio). Protein extracts were separated by SDSPAGE gels and transferred to PVDF membrane (ISEQ00010, Merck Millipore). The membranes were probed with indicated antibodies and visualized with the immobilon western chemiluminescent HRP substrate on ChemiDoc Touch chemiluminescence imaging system (Bio-Rad). The results were semi-quantitatively analyzed by Image Lab and Image J.

The working dilutions of antibodies in western blot: Anti-OPA1(1:1000), anti-Drp1(1:1000), anti-LDHA (1:1000), anti-MFF (1:1000), anti NF-κB p65 (1:1000), anti Phospho-NF-κB p65 (1:1000), anti-rabbit and anti-mouse IgG HRP-linked Antibody (1:3000)., anti-IL1 Receptor I/IL-1R-1 (1:200), anti-PFKP (1:2000), anti-PFKFB3 (1:1000), anti-Glucose Transporter-1 (GLUT1)(1:1000), anti-PKM2 (1:1000), anti-Hexokinase2 (HK2) (1:2000), anti-FIS1 (1:1000), anti-Phospho-IκB alpha (1:1000), anti-IκB alpha (1:1000), anti-IKK alpha/beta (1:1000), anti-MFN1 (1:1000), anti-MFN2 (1:1000) and anti-beta Actin (1:5000).

Cell proliferation experiments and Flow Cytometry

For cell proliferation experiments, $CD3^+$ T cells were labelled with CellTrace™ CFSE (Invitrogen),

were activated or not activated on a 24-well plate pre-coated with 5 μg/ml anti-CD3 in T cell culture medium supplemented with 2 μg/ml anti-CD28. CFSE dilution was assessed by flow cytometry on day 5 post-activation. All samples were acquired with BD LSRFortessa flow cytometer (Becton, Dickinson and Company) and analyzed with FlowJo software (TreeStar).

Imaging analysis of mitochondrial structure

MitoTracker (M7512, Thermo Fisher Scientific) and Hoechst 33342(C1025, Beyotime) staining were performed according to the manufacturer's instructions. For live imaging of mitochondria in cells, T cells were purified and activated as indicated, transferred to glass bottom dishes (801002, NEST) pre-coated with poly-D-lysine (C3012, Beyotime) in complete medium, and acquired using Olympus Confocal Microscope FV3000 with a 100× objective lens. Cells were kept in a humidified incubation chamber at 37°C with 5% CO_2 during acquisition. For nuclei visualization, Hoechst 33342 was added prior to acquisition. For the analysis of mitochondrial morphology in fixed cells, cells from the indicated genotypes were activated as indicated, stained with Mitotracker red 45min at 37 °C, allowed to settle for 30min at 37 °C into poly-D-lysine-coated coverslips, fixed 10min with 2.5% glutaraldehyde. At least 5-10 fields were randomly acquired per condition and biological replicate. Confocal images were analyzed using Image J [4]. Mean of mitochondria number, mean area and perimeter, and parameters representing the mitochondrial network (branches, branch lengths, branch junctions) were analyzed quantitatively.

For the mitochondria images captured by confocal microscopy, each group randomly selected more than 15 random fields of view from three or more biological replicates. Each field of view includes several cells, with a total cell count of over 100 across all fields of view. The mean mitochondria in cells and mean mitochondrial area in cells refers to the average number and average area divided by the

number cells in the field. Mean area and perimeter represent the area and perimeter averaged to a single mitochondrion in the field. The network structure of mitochondria is evaluated based on the number of branches, average branch length, and number of branch junctions' points.

For transmission electron microscopy (TEM), cells were fixed in Gluta fixative (for electron microscopy, 2.5% glutaraldehyde). Following fixation, the fixed cells were centrifuged. The 0.1 M PB (pH7.4) was added into the tube after supernatant was discarded, and then the precipitation was resuspended and washed in PBS for 3times, 3min each. The 1% agarose solution was prepared by heating and dissolving in advance. After being cooled, the agarose solution was added into the EP tube. Before agarose solidification, the precipitation was suspended with forceps and wrapped in the agarose. Agarose blocks with samples avoid light post fixed with 1% OsO_4 in 0.1 M PBS for 2h at room temperature. After remove OsO_4, the tissues are rinsed in 0.1 M PBS for 3 times, 15min each. Dehydrate at room temperature, Resin penetration and embedding, pour the pure EMBed 812 into the embedding models and insert the tissues into the pure EMBed. The embedding models with resin and samples were moved into 65°C ovens to polymerize for more than 48h. The resin blocks were cut into 60-80nm thin on the ultra-microtome, and the tissues were fished out onto the 150 meshes cuprum grids with formvar film. 2% uranium acetate saturated alcohol solution avoid light staining for 8 min, rinsed in 70% ethanol for 3 times and then rinsed in ultra-pure water for 3 times. 2.6% Lead citrate avoid CO_2 staining for 8 min, and then rinsed with ultra-pure water for 3 times. After dried by the filer paper, the cuprum grids were put into the grids board and dried overnight at room temperature. The cuprum grids are observed under Transmission Electron Microscope (HT7800/HT7700; HITACHI).

Metabolic assays

Seahorse XFe96 Extracellular Flux Analyzer was used to perform extracellular acidification rate (ECAR) and oxygen consumption rate (OCR) measurements. The Seahorse XF ATP rate or Glycolytic Rate Assay Kit (Agilent Technologies) was used, according to the manufacturer's instructions. All materials and compounds were obtained from Agilent Technologies.

The specific protocol is as follows: On the day before the experiment, the probe plate was hydrated, and more than 20 mL of detection solution was prepared in centrifuge tubes and left overnight in a non-CO_2 incubator with the probe plate. On the day of the experiment, the culture medium was prepared in advance by mixing 97 mL of medium with 1 mL each of glutamine, glucose, and pyruvate, all sourced from Agilent. T cells were seeded at a density of 0.4-3.0×10^5 cells per well in the XF96 cell culture microplate, which was pre-coated with poly-D-lysine and pre-incubated in a 37°C incubator for 2 hours. Cells were then incubated at 37°C in a non-CO_2 incubator for 25 minutes while the assay kit reagents, including Rot/AA, 2-DG, or oligomycin, were prepared. After incubation, T cell adhesion and density were observed under a microscope, and each well was replenished with 180 μL of culture medium. The plate was then centrifuged at 200g for 1 minute in a horizontal centrifuge with gradual acceleration and deceleration. Following this, the plate was returned to the 37°C non-CO_2 incubator for an additional 20-30 minutes before analysis. ECAR and OCR were measured during the basal stage (basal glycolysis + mitochondrial acidification), in response to Rot/AA (inhibitors of the mitochondrial electron transport chain, inducing compensatory glycolysis), and post-2-deoxy-D-glucose (a glucose analog) acidification. The basal and compensatory glycolytic rates were calculated using the Seahorse Glycolytic Rate Assay Report Generator, and account for contribution of CO_2 to extracellular acidification derived from mitochondrial respiration. The intracellular NAD^+/NADH levels were measured using the NAD^+/NADH assay kit with WST-8 (#S0175, Beyotime, China) according to the manufacturer's instructions. 5×10^6

cells were harvested by centrifugation at 200 g for 5 minutes at 4 °C, and the medium was subsequently removed. The cells were lysed in 200 μL of pre-cooled NAD^{+}/NADH lysis buffer, followed by centrifugation at 12,000 g for 10 minutes at 4 °C. Subsequently, 50-100 μL of the supernatant was transferred to a new tube and incubated at 60 °C for 30 minutes, followed by centrifugation at 10,000 g for 5 minutes at 4 °C. A 20 μL aliquot of the supernatant was then transferred to 96-well plates, incubated for 10 minutes at 37 °C, and followed by the addition of 10 μL of the color-developing solution. The NAD^{+}/NADH ratio was determined by measuring the absorbance of the mixture at 450 nm [5].

Cell transfection

RiboBio (Guangzhou, China) chemically synthesized siRNAs. A negative control siRNA (siRNA-NC) with random sequences targeting unknown mammalian genes was used. Transfection was carried out using Lipofectamine 3000 (L3000075, Thermo Fisher Scientific) for 8 h, and then the medium was changed by half. The efficacy was routinely checked by RT-qPCR 4d after transfection, cells were collected and used for indicated assays.

siRNA number	name	target sequence
siOPA1-1	genOFFTM st-h-OPA1_001	CCAGCAAGATTAGCTACGA
siOPA1-2	genOFFTM st-h-OPA1_002	GGGAAAGAGTATCAACTCA
siOPA1-3	genOFFTM st-h-OPA1_003	GCAGTATTGTTACAGACTT

Bioinformatics

The search for promoter sequences of human genes was carried out in the Eukaryotic Promoter Database (EPD) (database URL: https://epd.expasy.org/epd/ accessed by the 1 October 2023).

Pathways associated with the IL-1β were searched using the PathCards database of human biological pathways (pathway unification database) (database URL: http://pathcards.genecards.org/ accessed on 1 October 2023). Predictive analysis for the binding sites between RELA (p65) and the promoter region of OPA1 was conducted using humanTFDB, Unibind, and HOCOMOCO databases [6-8].

Chromatin immunoprecipitation assay (ChIP)

The ChIP assay was performed using the ChIP Assay Kit (Beyotime, China) following the manufacturer's guidelines. Briefly, CRC cells were cross-linked with 37%formaldehyde solution for 10min at room temperature and quenched with 125mM glycine. DNA fragments ranging from 200 to 500 bp were obtained by ultrasonication. Then the lysate was immunoprecipitated with anti-RELA (p65) or IgG antibodies. Immunoprecipitated DNAs were analyzed by RT-qPCR. Four hypothesized RELA (p65) binding sites (P1-P4) within the OPA1 promoter were selected, and corresponding primers were designed and synthesized, the primers were:

primer	5'to3'	3'to5'	Promoter region
P4	AAATCGGAAGCAAGAGGGC	CGGAAACAACCACTTCACCCT	chr3:193593058-193593367
P3	CCGTGAGAGGCGATGGATTG	GACTTCCGCAAGAGCCTGAC	chr3:193593028-193593278
P2	GAGATCTGGGCCTGTCCAAC	TTTGGCTCTCTGGCCATCAC	chr3:193592115-193592538

P1	GGGAGCCATCAAAGAAGCCT	CCTCCACCAGCTCCCTATTG	chr3:193591093-193591428

Statistical analysis

Statistical analyses were performed using SPSS 24.0 (USA) and Microsoft Excel. Statistical significance was set at $p < 0.05$. Data are presented as mean ± SEM. (standard error of the mean). Comparisons for two groups were calculated using unpaired two-tailed Student’s t-tests (for two groups meeting the normal distribution criteria, according to the Shapiro-Wilk normality or Kolmogorov-Smirnov test) and Mann Whitney test (for two groups that do not meet the normal distribution criteria). Comparisons of more than two groups and grouped data were calculated using one-way or two-way ANOVA (for groups meeting gaussian distribution) and corrected for multiple comparisons between groups or to a reference group using Dunnett’s tests, or Kruskal-Walli’s test (for groups do not meet gaussian distribution), Brown-Forsythe and Welch ANOVA tests were selected for ANOVA when the variance was not homogeneous. No statistical method was used to predetermine sample size, but a minimum of three samples were used per experimental group or condition. Experiments were not randomized.

References

[1] Fang J, Wang C, Shen C, Shan J, Wang X, Liu L, et al. The expression of CXCL10/CXCR3 and effect of the axis on the function of T lymphocyte involved in oral lichen planus. Inflammation 2019;42(3):799-810. doi: 10.1007/s10753-018-0934-0.

[2] Park H, Hurwitz S, Woo S. Oral lichen planus: REU scoring system correlates with pain. Oral Surgery, Oral Medicine, Oral Pathology and Oral Radiology 2012;114(1):75-82. doi:

10.1016/j.oooo.2012.02.013.

[3] Y. U. Jing-ting; M. Huan-xin; L. Kai-ning. An modified culture method of primary human gingival epithelial cells. Journal of Peking University(Health Sciences) 2016;48(4):733-737. doi: 10.3969/j.issn.1671-167X.2016.04.033.

[4] Chaudhry A, Shi R, Luciani DS. A pipeline for multidimensional confocal analysis of mitochondrial morphology, function, and dynamics in pancreatic β-cells. Am J Physiol Endocrinol Metab 2020;318(2):E87-E101. doi: 10.1152/ajpendo.00457.2019.

[5] Xia J, Zhang J, Wu X, Du W, Zhu Y, Liu X, et al. Blocking glycine utilization inhibits multiple myeloma progression by disrupting glutathione balance. Nat Commun 2022;13:4007.

[6] Puig RR, Boddie P, Khan A, Castro-Mondragon JA, Mathelier A. Unibind: maps of high-confidence direct TF-DNA interactions across nine species. Bmc Genomics 2021;22(1):482. doi: 10.1186/s12864-021-07760-6.

[7] Vorontsov IE, Eliseeva IA, Zinkevich A, Nikonov M, Abramov S, Boytsov A, et al. HOCOMOCO in 2024: a rebuild of the curated collection of binding models for human and mouse transcription factors. Nucleic Acids Res 2024;52(D1):D154-D163. doi: 10.1093/nar/gkad1077.

[8] Shen W, Chen S, Gan Z, Zhang Y, Yue T, Chen M, et al. AnimalTFDB 4.0: a comprehensive animal transcription factor database updated with variation and expression annotations. Nucleic Acids Res 2023;51(D1):D39-D45. doi: 10.1093/nar/gkac907.

supplementary tables 1-3

Table S1. OLP patient clinical data information

Case no.	Sex	Age (years)	Affected sites	Clinical form	The Scores of disease activity
1	M	29	Cheek	Erosive	6
2	M	41	Cheek	Erosive	11
3	F	42	Cheek	Erosive	13
4	M	75	Check	Erosive	4
5	F	61	Check, Lip	Erosive	22.5
6	M	23	Cheek	Erosive	9
7	F	42	Cheek, Gingiva	Erosive	17.5
8	M	53	Tongue	Erosive	5.5
9	M	30	Check	Erosive	11
10	M	25	Cheek	Erosive	7.5
11	F	27	Cheek, Tongue	Erosive	15
12	M	38	Cheek, Lip	Erosive	8
13	M	37	Check, Lip	Erosive	26.5
14	M	30	Check, Tongue, Lip	Erosive	22
15	F	55	Cheek, Lip	Erosive	12
16	F	60	Cheek, Gingiva, Tongue, Lip	Non-erosive	6
17	F	34	Check, Tongue	Non-erosive	3
18	F	63	Cheek, Gingiva, Tongue	Non-erosive	6
19	M	34	Check, Gingiva, Tongue	Non-erosive	5
20	M	24	Check, Tongue	Non-erosive	3
21	F	44	Cheek	Non-erosive	2
22	M	21	Cheek, Tongue	Non-erosive	4
23	F	32	Check, Gingiva	Non-erosive	4
24	M	44	Cheek, Gingiva, Tongue	Non-erosive	5
25	M	23	Check, Gingiva, Tongue	Non-erosive	6
26	M	33	Cheek, Tongue	Non-erosive	4
27	M	44	Cheek, Gingiva, Tongue	Non-erosive	5
28	F	30	Cheek, Gingiva, Tongue	Non-erosive	5
29	M	36	Cheek, Tongue	Non-erosive	3
30	M	33	Cheek, Tongue, Lip	Non-erosive	4

Table S2. OLP erosive/non-erosive patient clinical parameter scoring sheet

Characteristic		Non-erosive group (n = 15)	Erosive group (n = 15)	*p*
Gender	Male	9	10	
	Female	6	5	1
Age	< 40	10	8	
	> 40	5	7	0.71
Disease course	< 6 months	11	7	
	≥ 6 months	4	8	0.264
Disease score (mean ± SD)		4.33 ± 1.23	12.70 ± 6.78	< 0.001***

OLP, oral lichen planus; M, male; F, female. Difference was considered significant at $P < 0.05$

Table S3. Predicted transcription factors binding to the OPA1 promoter region through humanTFDB.

TF	source	sequence	start	stop	strand	score	p-value	q-value
RELA	hTFtarget	NC_000003.12:193590208-193593207	2903	2916	+	21.8158	2.07E-08	0.000123
RELA	hTFtarget	NC_000003.12:193590208-193593207	2901	2921	+	21.4868	4.10E-08	0.000244
RELA	hTFtarget	NC_000003.12:193590208-193593207	2905	2916	+	21.2	6.23E-08	0.000372
RELA	hTFtarget	NC_000003.12:193590208-193593207	2905	2916	+	21.0921	8.78E-08	0.000525
RELA	hTFtarget	NC_000003.12:193590208-193593207	2903	2916	+	20.6316	4.42E-08	0.000264
RELA	hTFtarget	NC_000003.12:193590208-193593207	2906	2917	+	19.9848	1.64E-07	0.000979
RELA	hTFtarget	NC_000003.12:193590208-193593207	2905	2916	+	19.6184	8.78E-08	0.000525
RELA	hTFtarget	NC_000003.12:193590208-193593207	2905	2916	+	19.0263	1.60E-07	0.000956
RELA	hTFtarget	NC_000003.12:193590208-193593207	2903	2918	+	19.0263	2.61E-07	0.00156
RELA	hTFtarget	NC_000003.12:193590208-193593207	2905	2916	+	18.9211	3.07E-07	0.00183
RELA	hTFtarget	NC_000003.12:193590208-193593207	2905	2916	+	18.6447	3.76E-07	0.00225
RELA	hTFtarget	NC_000003.12:193590208-193593207	2906	2917	+	18.4394	5.09E-07	0.00267
FOS	hTFtarget	NC_000003.12:193590208-193593207	2849	2860	+	18.25	3.44E-07	0.00202
FOS	hTFtarget	NC_000003.12:193590208-193593207	2849	2860	+	17.5658	6.73E-07	0.00392
FOS	hTFtarget	NC_000003.12:193590208-193593207	46	60	+	17.1667	5.37E-07	0.00321
FOS	hTFtarget	NC_000003.12:193590208-193593207	2849	2860	+	17.1316	7.09E-07	0.00416
RELA	hTFtarget	NC_000003.12:193590208-193593207	2908	2916	+	16.8684	2.23E-06	0.00668
RELA	hTFtarget	NC_000003.12:193590208-193593207	2905	2920	+	16.0571	1.86E-06	0.00556
RELA	hTFtarget	NC_000003.12:193590208-193593207	2906	2917	+	15.9429	2.58E-06	0.0077
MEF2A	database	NC_000003.12:193590208-193593207	1135	1146	+	15.9293	3.65E-06	0.0213

RELA	hTFtarget	NC_000003.12:193590208-193593207	2201	2216	+	15.6842	2.53E-06	0.0149
MYC	hTFtarget	NC_000003.12:193590208-193593207	47	58	+	15.6711	3.01E-06	0.0179
JUN	hTFtarget	NC_000003.12:193590208-193593207	134	142	+	15.6515	4.91E-06	0.0289
JUN	hTFtarget	NC_000003.12:193590208-193593207	134	142	+	15.6515	4.91E-06	0.0287
FOS	hTFtarget	NC_000003.12:193590208-193593207	1935	1946	+	15.5263	3.42E-06	0.01
RELA	hTFtarget	NC_000003.12:193590208-193593207	2907	2918	+	15.3857	3.64E-06	0.0109
MEF2A	hTFtarget	NC_000003.12:193590208-193593207	998	1011	+	15.3433	2.65E-06	0.0153
JUN	hTFtarget	NC_000003.12:193590208-193593207	130	145	+	15.1818	5.44E-06	0.0324
MYC	hTFtarget	NC_000003.12:193590208-193593207	2136	2147	+	14.9868	4.51E-06	0.0268
FOS	hTFtarget	NC_000003.12:193590208-193593207	47	58	+	14.9697	3.90E-06	0.0233
RELA	hTFtarget	NC_000003.12:193590208-193593207	2201	2216	+	14.9429	2.22E-06	0.0132
MYC	hTFtarget	NC_000003.12:193590208-193593207	2844	2859	+	14.9342	4.74E-06	0.0276
STAT1	hTFtarget	NC_000003.12:193590208-193593207	134	142	+	14.8289	4.91E-06	0.0293
FOS	hTFtarget	NC_000003.12:193590208-193593207	134	144	+	14.7273	8.71E-06	0.0511
RELA	hTFtarget	NC_000003.12:193590208-193593207	134	142	+	14.6711	4.91E-06	0.0294
FOS	hTFtarget	NC_000003.12:193590208-193593207	134	142	+	14.6364	1.22E-05	0.0714
RELA	hTFtarget	NC_000003.12:193590208-193593207	2905	2920	+	14.5857	3.28E-06	0.0098
ATF2	hTFtarget	NC_000003.12:193590208-193593207	48	59	+	14.5658	6.01E-06	0.0356
FOS	hTFtarget	NC_000003.12:193590208-193593207	1935	1946	+	14.5	6.66E-06	0.0194
MAX	hTFtarget	NC_000003.12:193590208-193593207	134	142	+	14.4737	8.94E-06	0.0532
FOS	hTFtarget	NC_000003.12:193590208-193593207	47	59	+	14.2	4.75E-06	0.0284
JUN	hTFtarget	NC_000003.12:193590208-193593207	134	142	+	14.0714	8.94E-06	0.053

JUN	database	NC_000003.12:193590208-193593207	134	142	+	14.069	8.94E-06	0.0535
FOS	hTFtarget	NC_000003.12:193590208-193593207	50	61	+	14	3.70E-06	0.0221
STAT1	hTFtarget	NC_000003.12:193590208-193593207	1936	1944	+	13.8816	1.46E-05	0.0426
FOS	database	NC_000003.12:193590208-193593207	47	58	+	13.8305	1.58E-06	0.00934
JUN	hTFtarget	NC_000003.12:193590208-193593207	134	145	+	13.8182	1.42E-05	0.0682
FOS	hTFtarget	NC_000003.12:193590208-193593207	134	142	+	13.6	1.22E-05	0.0728
STAT1	hTFtarget	NC_000003.12:193590208-193593207	2096	2107	+	13.5286	1.78E-06	0.0103
MEF2A	hTFtarget	NC_000003.12:193590208-193593207	134	142	+	13.4211	2.85E-05	0.0848
FOS	hTFtarget	NC_000003.12:193590208-193593207	1935	1946	+	13.3947	1.43E-05	0.0419
JUN	hTFtarget	NC_000003.12:193590208-193593207	79	87	+	13.3944	1.22E-05	0.071
STAT1	hTFtarget	NC_000003.12:193590208-193593207	2780	2801	+	13.3421	7.43E-06	0.0443
STAT1	hTFtarget	NC_000003.12:193590208-193593207	2787	2802	+	13.2632	6.71E-06	0.0399
JUN	hTFtarget	NC_000003.12:193590208-193593207	134	142	+	13.2143	2.52E-05	0.0754
RELA	hTFtarget	NC_000003.12:193590208-193593207	2782	2797	+	13.2105	1.53E-05	0.045
MYC	hTFtarget	NC_000003.12:193590208-193593207	2789	2797	+	13.1711	1.70E-05	0.033
MEF2A	database	NC_000003.12:193590208-193593207	1134	1147	+	13.1429	1.63E-05	0.0543
FOS	hTFtarget	NC_000003.12:193590208-193593207	132	143	+	13.0758	2.35E-05	0.14
MEF2A	hTFtarget	NC_000003.12:193590208-193593207	1135	1148	+	13.0597	1.70E-05	0.0489
MAX	hTFtarget	NC_000003.12:193590208-193593207	2251	2262	+	12.974	2.05E-05	0.0816
MEF2A	database	NC_000003.12:193590208-193593207	997	1010	+	12.9221	1.88E-05	0.0543
STAT1	hTFtarget	NC_000003.12:193590208-193593207	2764	2775	+	12.9079	2.40E-05	0.0683
JUN	database	NC_000003.12:193590208-193593207	129	142	+	12.9038	2.82E-05	0.0828

JUN	hTFtarget	NC_000003.12:193590208-193593207	132	143	+	12.8636	2.55E-05	0.152
STAT1	hTFtarget	NC_000003.12:193590208-193593207	833	844	+	12.8289	2.19E-05	0.13
FOS	hTFtarget	NC_000003.12:193590208-193593207	133	144	+	12.803	3.08E-05	0.092
MEF2A	hTFtarget	NC_000003.12:193590208-193593207	134	142	+	12.8	2.85E-05	0.0666
FOS	hTFtarget	NC_000003.12:193590208-193593207	133	144	+	12.7727	2.70E-05	0.161
MEF2A	hTFtarget	NC_000003.12:193590208-193593207	996	1011	+	12.6866	2.13E-05	0.065
RELA	hTFtarget	NC_000003.12:193590208-193593207	1691	1697	+	12.6579	5.35E-05	0.317
ATF2	hTFtarget	NC_000003.12:193590208-193593207	2429	2437	+	12.6447	3.08E-05	0.184
MYC	hTFtarget	NC_000003.12:193590208-193593207	134	142	+	12.6447	3.99E-05	0.119
MYC	hTFtarget	NC_000003.12:193590208-193593207	134	142	+	12.6447	3.99E-05	0.119
MEF2A	hTFtarget	NC_000003.12:193590208-193593207	1133	1148	+	12.6119	2.23E-05	0.065
RELA	hTFtarget	NC_000003.12:193590208-193593207	127	135	+	12.6053	3.59E-05	0.107
JUN	hTFtarget	NC_000003.12:193590208-193593207	127	135	+	12.6	4.83E-05	0.0955
FOS	hTFtarget	NC_000003.12:193590208-193593207	127	135	+	12.5286	4.97E-05	0.143
AP1	database	NC_000003.12:193590208-193593207	134	142	+	12.5135	2.62E-05	0.116
STAT1	hTFtarget	NC_000003.12:193590208-193593207	2211	2222	+	12.5	3.05E-05	0.182
MEF2A	hTFtarget	NC_000003.12:193590208-193593207	2431	2437	+	12.4868	6.51E-05	0.388
JUN	hTFtarget	NC_000003.12:193590208-193593207	132	147	+	12.4091	2.70E-05	0.0805
JUN	database	NC_000003.12:193590208-193593207	127	135	+	12.3621	4.32E-05	0.129
FOS	hTFtarget	NC_000003.12:193590208-193593207	134	142	+	12.3571	5.06E-05	0.1
MYC	hTFtarget	NC_000003.12:193590208-193593207	134	142	+	12.3553	5.37E-05	0.107
JUN	hTFtarget	NC_000003.12:193590208-193593207	134	142	+	12.3485	2.85E-05	0.0853

MAX	hTFtarget	NC_000003.12:193590208-193593207	127	135	+	12.1184	6.06E-05	0.12
ATF2	hTFtarget	NC_000003.12:193590208-193593207	2312	2323	+	12.1053	3.60E-05	0.106
MYC	hTFtarget	NC_000003.12:193590208-193593207	2587	2595	+	12.1039	1.89E-05	0.0777
JUN	hTFtarget	NC_000003.12:193590208-193593207	444	452	+	11.9296	5.53E-05	0.16
FOS	hTFtarget	NC_000003.12:193590208-193593207	134	142	+	11.9143	7.74E-05	0.154
AP1	database	NC_000003.12:193590208-193593207	128	135	+	11.8947	1.79E-05	0.0532
AP1	database	NC_000003.12:193590208-193593207	135	142	+	11.8947	1.79E-05	0.0532
STAT1	hTFtarget	NC_000003.12:193590208-193593207	1948	1956	+	11.8553	5.48E-05	0.0801
RELA	hTFtarget	NC_000003.12:193590208-193593207	1691	1697	+	11.8143	5.35E-05	0.318
MEF2A	database	NC_000003.12:193590208-193593207	1134	1149	+	11.8	1.74E-05	0.0579
JUN	hTFtarget	NC_000003.12:193590208-193593207	127	135	+	11.7727	4.78E-05	0.14
MYC	hTFtarget	NC_000003.12:193590208-193593207	1935	1956	+	11.7632	2.47E-05	0.0632
STAT1	database	NC_000003.12:193590208-193593207	1876	1885	+	11.759	4.86E-05	0.289
RELA	hTFtarget	NC_000003.12:193590208-193593207	1952	1963	+	11.6974	4.89E-05	0.282
STAT1	hTFtarget	NC_000003.12:193590208-193593207	2759	2774	+	11.6761	4.54E-05	0.129
RELA	hTFtarget	NC_000003.12:193590208-193593207	2878	2886	+	11.6184	8.34E-05	0.496
FOS	hTFtarget	NC_000003.12:193590208-193593207	1691	1697	+	11.5571	5.35E-05	0.314
AP1	database	NC_000003.12:193590208-193593207	132	144	+	11.5556	4.41E-05	0.163
ELK1	database	NC_000003.12:193590208-193593207	2386	2395	+	11.5444	4.74E-05	0.283
FOS	hTFtarget	NC_000003.12:193590208-193593207	1691	1697	+	11.5429	5.35E-05	0.314
RELA	hTFtarget	NC_000003.12:193590208-193593207	364	375	+	11.4935	5.74E-05	0.164
FOS	hTFtarget	NC_000003.12:193590208-193593207	104	115	+	11.4868	3.80E-05	0.0744

MYC	hTFtarget	NC_000003.12:193590208-193593207	2878	2886	+	11.4868	9.06E-05	0.27
JUN	hTFtarget	NC_000003.12:193590208-193593207	134	142	+	11.4605	6.84E-05	0.202
RELA	hTFtarget	NC_000003.12:193590208-193593207	1952	1963	+	11.4211	6.06E-05	0.35
STAT1	hTFtarget	NC_000003.12:193590208-193593207	225	236	+	11.4	5.02E-05	0.0741
MEF2A	database	NC_000003.12:193590208-193593207	997	1012	+	11.3778	2.02E-05	0.0579
FOS	hTFtarget	NC_000003.12:193590208-193593207	134	142	+	11.3714	8.85E-05	0.176
STAT1	hTFtarget	NC_000003.12:193590208-193593207	2878	2886	+	11.3571	6.10E-05	0.363
JUN	hTFtarget	NC_000003.12:193590208-193593207	134	142	+	11.303	4.08E-05	0.0813
FOS	hTFtarget	NC_000003.12:193590208-193593207	104	115	+	11.2895	4.27E-05	0.0828
STAT1	hTFtarget	NC_000003.12:193590208-193593207	2850	2858	+	11.2632	8.11E-05	0.0948
MAX	hTFtarget	NC_000003.12:193590208-193593207	2095	2110	+	11.2468	5.24E-05	0.244
MYC	hTFtarget	NC_000003.12:193590208-193593207	1540	1555	+	11.2308	6.35E-05	0.123
MEF2D	database	NC_000003.12:193590208-193593207	1136	1147	+	11.1786	5.83E-05	0.292
RELA	hTFtarget	NC_000003.12:193590208-193593207	1475	1486	+	11.1579	7.17E-05	0.246
MAX	hTFtarget	NC_000003.12:193590208-193593207	609	620	+	11.1429	7.94E-05	0.151
STAT1	hTFtarget	NC_000003.12:193590208-193593207	2783	2798	+	11.0986	6.74E-05	0.129
JUN	hTFtarget	NC_000003.12:193590208-193593207	132	146	+	11.0526	4.23E-05	0.126
STAT1	hTFtarget	NC_000003.12:193590208-193593207	1874	1885	+	11.026	8.40E-05	0.496
STAT1	hTFtarget	NC_000003.12:193590208-193593207	1541	1552	+	11.0132	8.76E-05	0.101
STAT1	hTFtarget	NC_000003.12:193590208-193593207	2764	2779	+	10.9718	7.15E-05	0.2
MYC	hTFtarget	NC_000003.12:193590208-193593207	2952	2967	+	10.9605	7.01E-05	0.185
RELA	hTFtarget	NC_000003.12:193590208-193593207	2886	2894	+	10.9211	5.19E-05	0.309

ELK1	hTFtarget	NC_000003.12:193590208-193593207	2047	2058	+	10.9211	7.39E-05	0.225
FOS	database	NC_000003.12:193590208-193593207	2311	2322	+	10.8983	8.19E-05	0.243
MAX	hTFtarget	NC_000003.12:193590208-193593207	2787	2798	+	10.8816	3.74E-05	0.0553
RELA	hTFtarget	NC_000003.12:193590208-193593207	1879	1890	+	10.8816	8.55E-05	0.246
FOS	hTFtarget	NC_000003.12:193590208-193593207	132	143	+	10.8788	7.36E-05	0.219
RELA	hTFtarget	NC_000003.12:193590208-193593207	2765	2780	+	10.8553	6.81E-05	0.1
FOS	hTFtarget	NC_000003.12:193590208-193593207	127	135	+	10.8182	9.18E-05	0.268
STAT1	hTFtarget	NC_000003.12:193590208-193593207	1877	1888	+	10.8	9.69E-05	0.094
STAT1	hTFtarget	NC_000003.12:193590208-193593207	2094	2105	+	10.7763	7.33E-05	0.144
MYC	hTFtarget	NC_000003.12:193590208-193593207	1948	1963	+	10.75	8.08E-05	0.185
MYC	hTFtarget	NC_000003.12:193590208-193593207	1071	1086	+	10.6974	4.59E-05	0.216
MEF2C	database	NC_000003.12:193590208-193593207	1630	1644	+	10.678	7.84E-05	0.115
RELA	hTFtarget	NC_000003.12:193590208-193593207	1879	1894	+	10.6667	8.40E-05	0.481
MEF2A	hTFtarget	NC_000003.12:193590208-193593207	1631	1646	+	10.6567	9.46E-05	0.183
MEF2C	hTFtarget	NC_000003.12:193590208-193593207	1630	1644	+	10.6119	9.81E-05	0.14
ATF2	hTFtarget	NC_000003.12:193590208-193593207	1581	1592	+	10.6053	9.21E-05	0.524
STAT1	hTFtarget	NC_000003.12:193590208-193593207	1071	1077	+	10.5857	5.35E-05	0.286
MAX	hTFtarget	NC_000003.12:193590208-193593207	279	294	+	10.5658	8.68E-05	0.283
MYC	hTFtarget	NC_000003.12:193590208-193593207	2842	2856	+	10.5132	4.12E-05	0.123
MYC	hTFtarget	NC_000003.12:193590208-193593207	2786	2797	+	10.4868	9.77E-05	0.576
MYC	hTFtarget	NC_000003.12:193590208-193593207	2785	2800	+	10.4605	3.55E-05	0.211
MYC	hTFtarget	NC_000003.12:193590208-193593207	2762	2773	+	10.4605	9.54E-05	0.279

STAT1	hTFtarget	NC_000003.12:193590208-193593207	2096	2107	+	10.4342	5.99E-05	0.171
MAX	hTFtarget	NC_000003.12:193590208-193593207	367	382	+	10.4156	7.90E-05	0.466
MYC	hTFtarget	NC_000003.12:193590208-193593207	2776	2797	+	10.4035	8.31E-05	0.238
STAT1	hTFtarget	NC_000003.12:193590208-193593207	132	147	+	10.3506	5.49E-05	0.327
FOS	hTFtarget	NC_000003.12:193590208-193593207	104	115	+	10.3421	7.67E-05	0.15
FOS	hTFtarget	NC_000003.12:193590208-193593207	132	143	+	10.2879	7.61E-05	0.227
RELA	hTFtarget	NC_000003.12:193590208-193593207	2788	2803	+	10.2632	9.49E-05	0.112
STAT1	database	NC_000003.12:193590208-193593207	1873	1894	+	10.197	9.90E-05	0.583
MYC	hTFtarget	NC_000003.12:193590208-193593207	2841	2862	+	10.1579	5.81E-05	0.0632
MYC	hTFtarget	NC_000003.12:193590208-193593207	2851	2872	+	10.1447	5.85E-05	0.0632
MEF2D	database	NC_000003.12:193590208-193593207	999	1010	+	10.1429	9.94E-05	0.292
FOS	hTFtarget	NC_000003.12:193590208-193593207	2771	2782	+	9.94737	7.57E-05	0.112
MYC	hTFtarget	NC_000003.12:193590208-193593207	447	455	+	9.88158	3.75E-05	0.0548
JUN	hTFtarget	NC_000003.12:193590208-193593207	133	144	+	9.86364	9.11E-05	0.272
MAX	hTFtarget	NC_000003.12:193590208-193593207	2788	2799	+	9.78947	5.97E-05	0.0704
STAT1	hTFtarget	NC_000003.12:193590208-193593207	1610	1627	+	9.73239	8.15E-05	0.467
MYC	hTFtarget	NC_000003.12:193590208-193593207	964	979	+	9.71053	7.43E-05	0.216
MYC	hTFtarget	NC_000003.12:193590208-193593207	100	121	+	9.61842	7.62E-05	0.0632
MYC	hTFtarget	NC_000003.12:193590208-193593207	2786	2801	+	9.53947	3.50E-05	0.209
MAX	hTFtarget	NC_000003.12:193590208-193593207	2770	2781	+	9.44737	6.98E-05	0.0825
RELA	hTFtarget	NC_000003.12:193590208-193593207	2907	2918	+	9.34286	3.18E-05	0.095
MYC	hTFtarget	NC_000003.12:193590208-193593207	2781	2796	+	9.28571	4.78E-05	0.0553

MYC	hTFtarget	NC_000003.12:193590208-193593207	2786	2801	+	9.21053	7.60E-05	0.226
JUN	hTFtarget	NC_000003.12:193590208-193593207	127	135	+	8.90909	8.86E-05	0.174
MYC	hTFtarget	NC_000003.12:193590208-193593207	2787	2798	+	8.90789	9.30E-05	0.136
MYC	hTFtarget	NC_000003.12:193590208-193593207	930	945	+	8.84211	5.40E-05	0.129
MAX	hTFtarget	NC_000003.12:193590208-193593207	2749	2760	+	8.57895	7.24E-05	0.117
FOS	hTFtarget	NC_000003.12:193590208-193593207	132	145	+	8.51515	7.75E-05	0.231
MYC	hTFtarget	NC_000003.12:193590208-193593207	2763	2778	+	8.38158	6.55E-05	0.129
RELA	hTFtarget	NC_000003.12:193590208-193593207	2197	2214	+	8.28947	9.00E-05	0.537
MAX	hTFtarget	NC_000003.12:193590208-193593207	2771	2782	+	8.23684	7.96E-05	0.117
MYC	hTFtarget	NC_000003.12:193590208-193593207	2231	2252	+	7.96053	1.74E-05	0.0536
MYC	hTFtarget	NC_000003.12:193590208-193593207	2842	2857	+	7.65789	7.32E-05	0.218
RELA	hTFtarget	NC_000003.12:193590208-193593207	837	851	+	6.57895	8.49E-05	0.273
STAT1	hTFtarget	NC_000003.12:193590208-193593207	2818	2833	+	5.60526	8.68E-05	0.517
STAT1	hTFtarget	NC_000003.12:193590208-193593207	2818	2833	+	5.60526	8.68E-05	0.518
STAT1	database	NC_000003.12:193590208-193593207	1870	1890	+	3.40789	9.06E-05	0.539
STAT1	hTFtarget	NC_000003.12:193590208-193593207	650	665	+	0.302632	8.10E-05	0.481

The higher the score, the greater the likelihood of predicted binding.
