# Supplementary figures and images for: Pyroptosis of oral keratinocyte contributes to energy metabolic reprogramming of T cells in oral lichen planus via OPA1-mediated mitochondrial fusion
Source: Cell Death Discov. 2024 Sep 17;10:408. doi: 10.1038/s41420-024-02174-1 (PMC11408637; doi:10.1038/s41420-024-02174-1)

Figure 3D

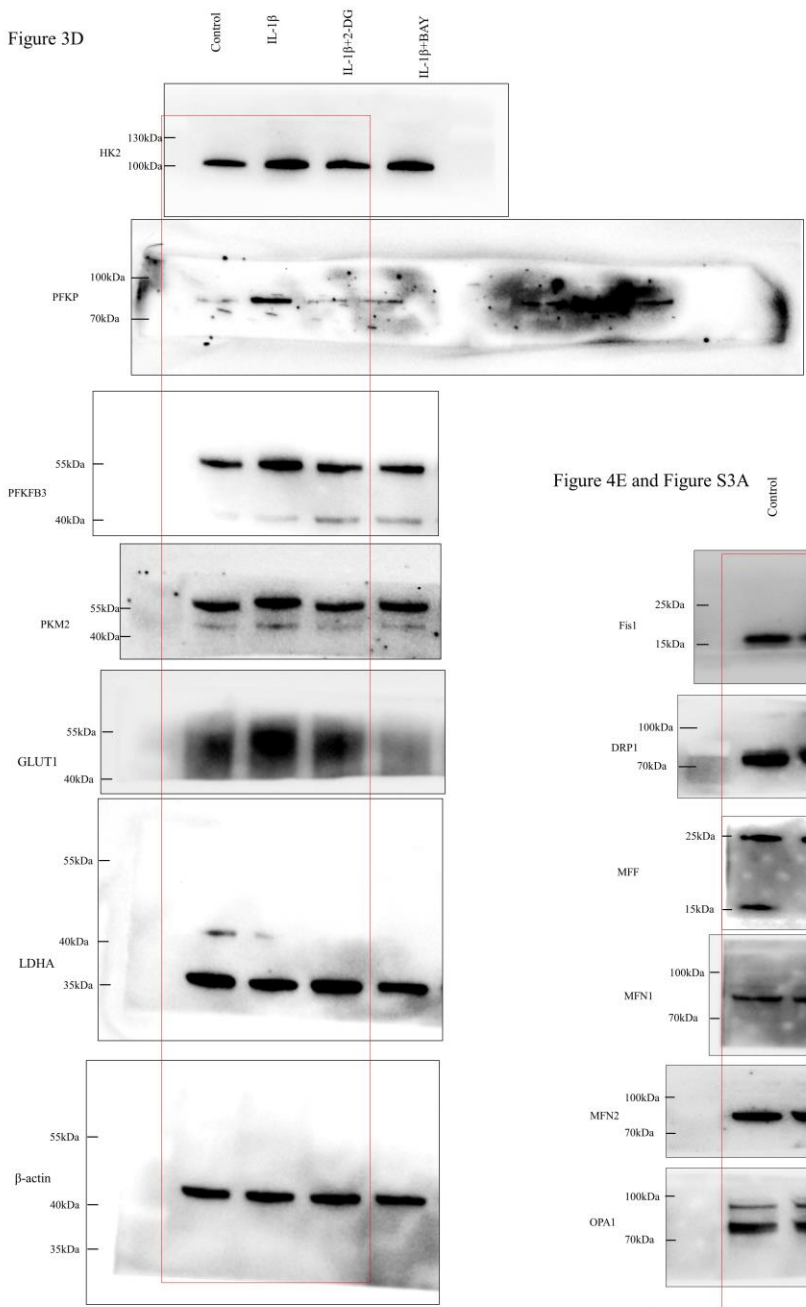

Figure 4E and Figure S3A

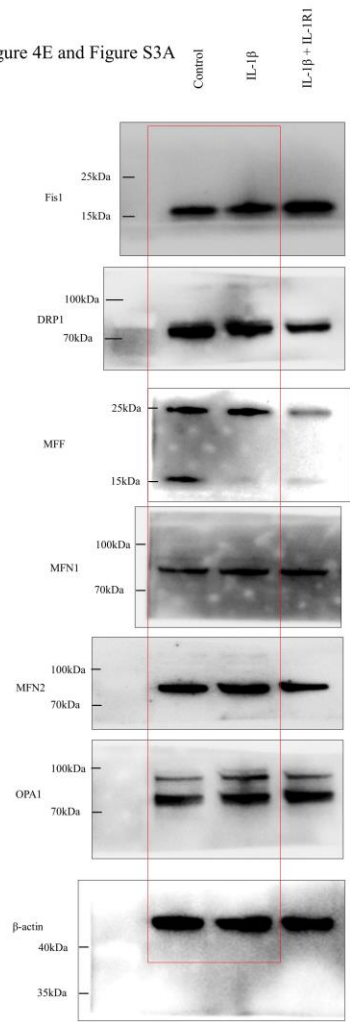

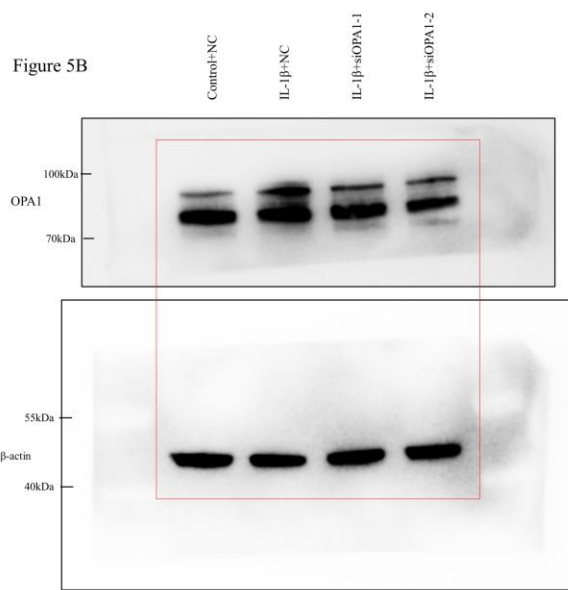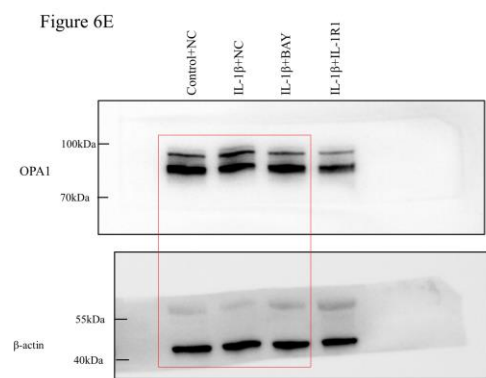

Figure 6B

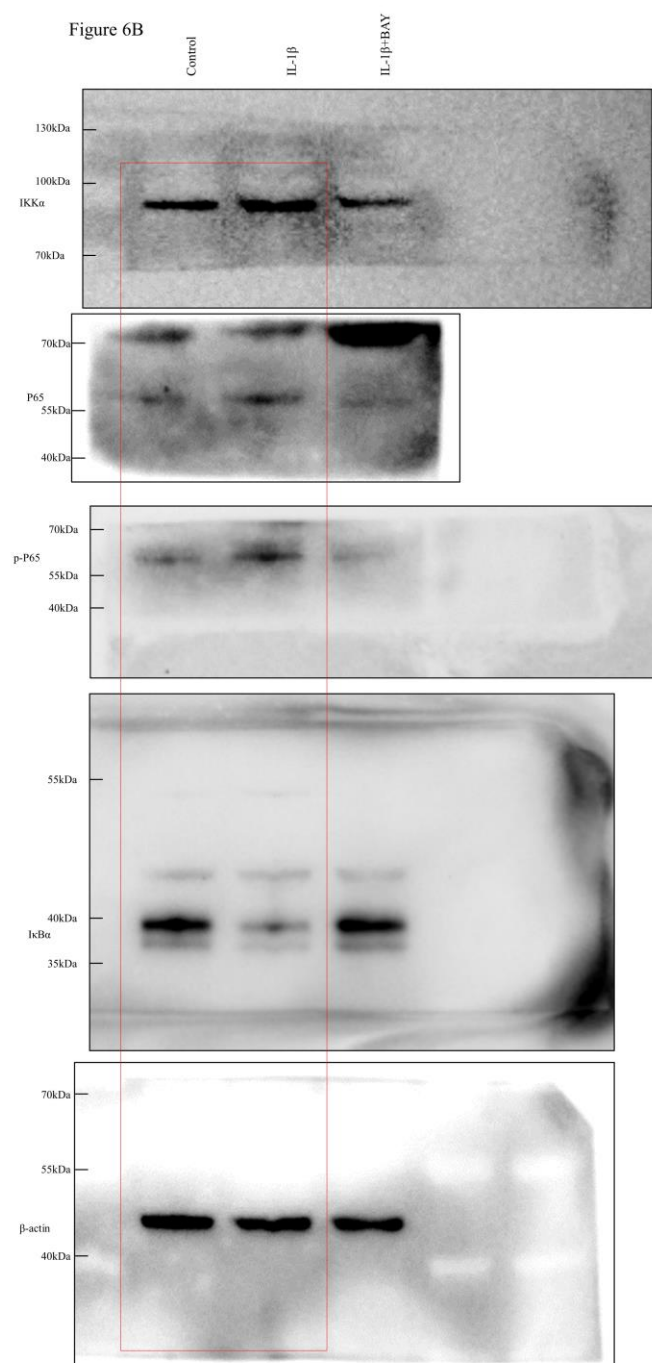

Figure S1B

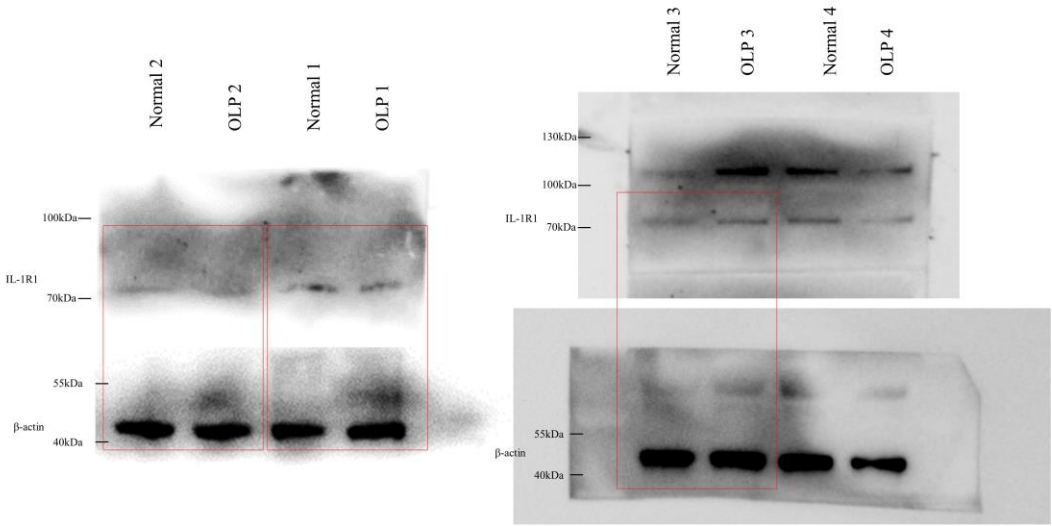

Figure S4C

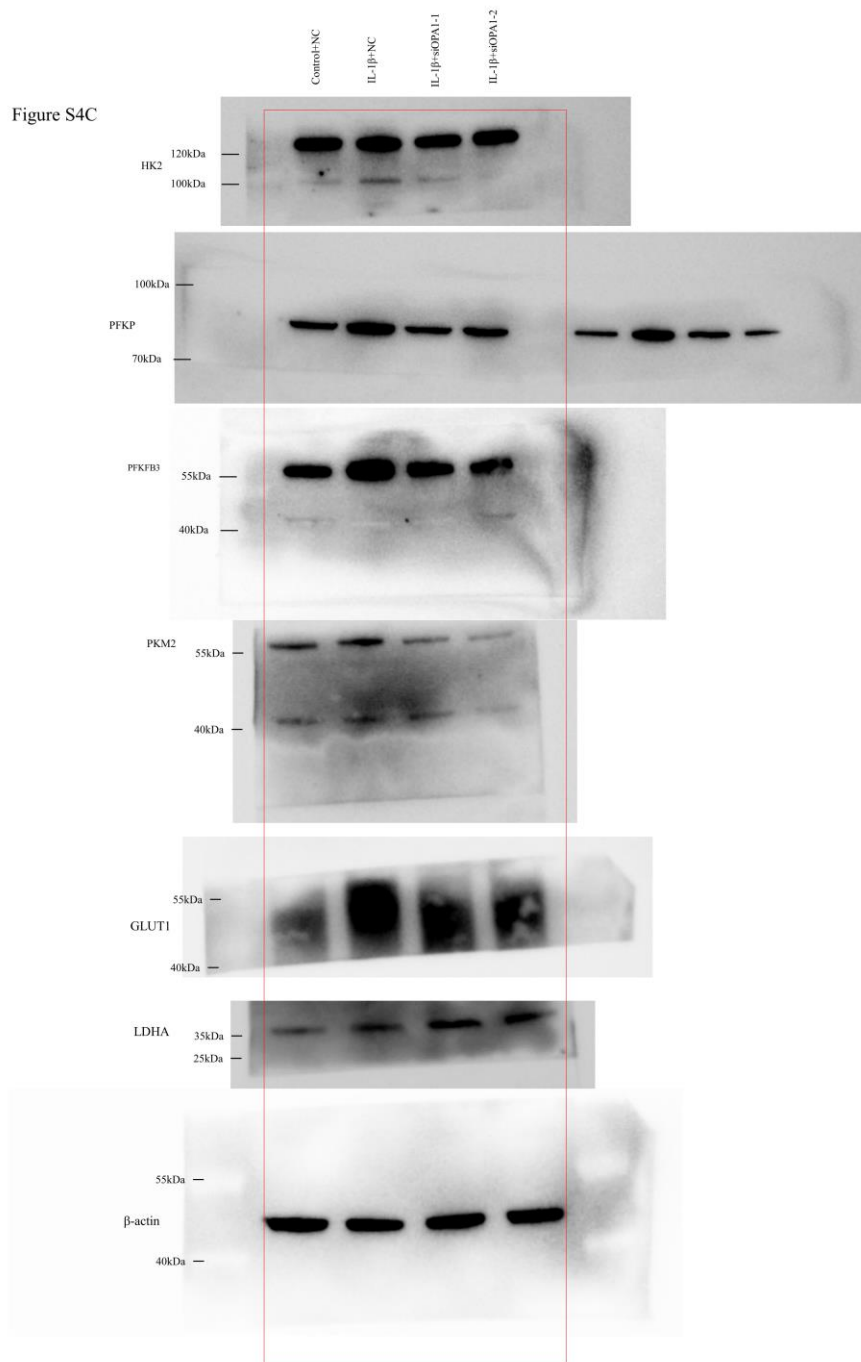

Figure S5E

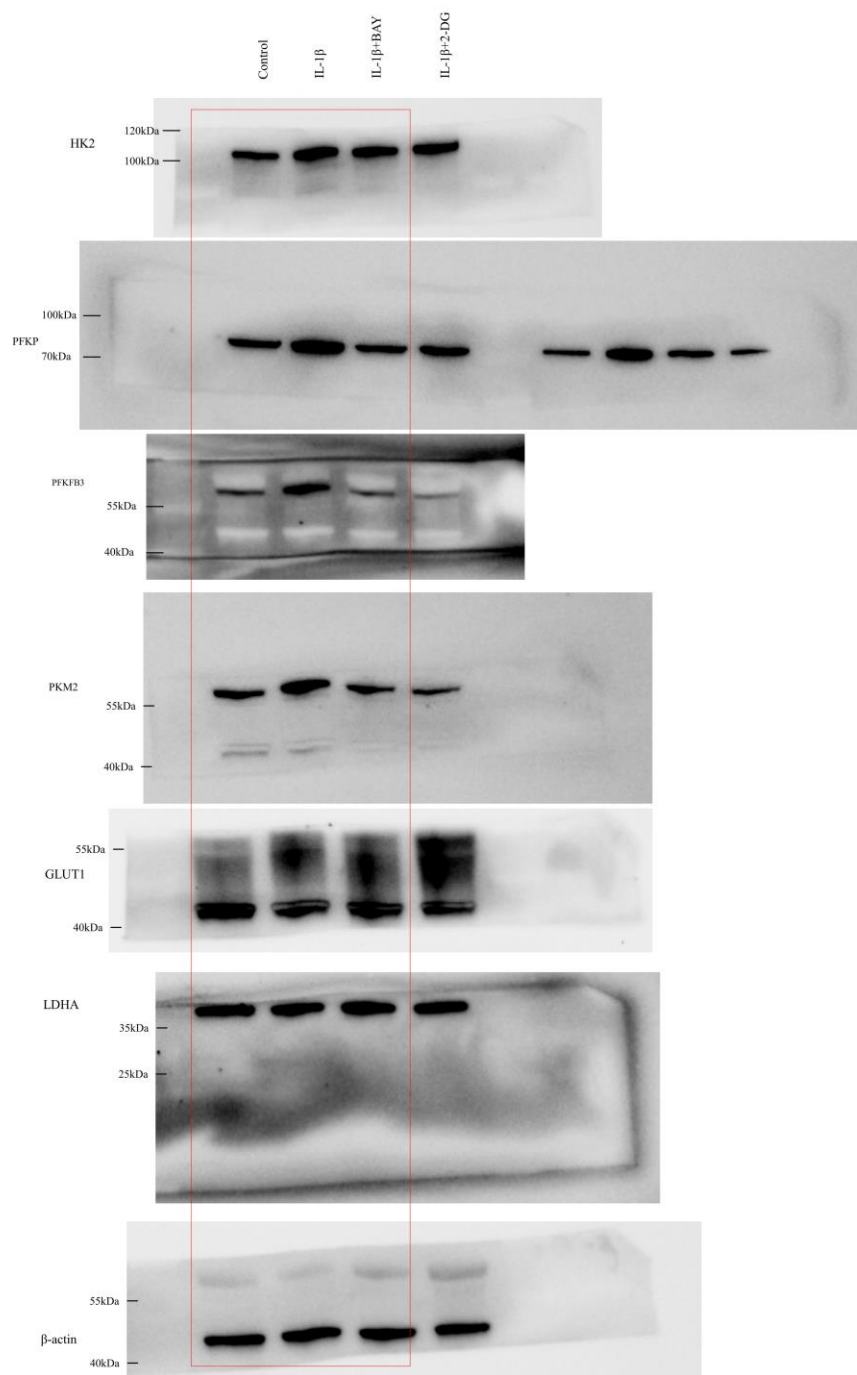

Supplement: Supplementary file 2 — original western blots [file 41420_2024_2174_MOESM2_ESM.pdf]
